# Supplementary material for: Mechanistic insights into ASO-RNA complexation: Advancing antisense oligonucleotide design strategies
Source: Mol Ther Nucleic Acids. 2024 Oct 4;35(4):102351. doi: 10.1016/j.omtn.2024.102351 (PMC11530825; doi:10.1016/j.omtn.2024.102351)
Supplement: Document S1. Figures S1–S15 and Table S1 [file mmc1.pdf]

OMTN, Volume *35*

Supplemental information

Mechanistic insights into ASO-RNA complexation: Advancing antisense oligonucleotide design strategies

Johanna Hörberg, Antonio Carlesso, and Anna Reymer

Table S1. List of FDA-approved antisense oligonucleotide (ASO) drugs.

**Name**	**Molecular target/tissue**	**Category/length**	**Approval Date**	**Indications**
Fomivirsen (Vitravene)	CMV IE-2/eye	ASO/21 mer ps DNA	1998.08	Cytomegalovirus Retinitis
Mipomersen (Kynamro)	ApoB-100/liver	ASO/20 mer gapmer, PS	2013.01	Homozygous Familial Hypercholesterolemia
Eleplinsen (Exondlys 51)	Dystrophin protein/muscle	ASO-SSO****/30 mer, DNA PMO*	2016.09	Duchenne Muscular Dystrophy
Nusinersen (Spinraza)	Survival motor neuron (SMN) protein/CNS**	ASO/18 mer, PS***	2016.12	Spinal Muscular Atrophy
Inotersen (Tegsedi)	TTR/liver	ASO/20 mer gapmer, PS	2018.01	Hereditary Transthyretin Amyloidosis, Polyneuropathy
Golodirsen (Vyondlys 53)	analogous to Eleplinsen (Exondlys 51) but instead skipping exon 53	ASO-SSO/25 mer DNA PMO	2019.12	Duchenne Muscular Dystrophy
Volanesorsen (Waylivra)	Apolipoprotein CIII /liver	ASO/20 mer gapmer, PS	2019	Familial Chylomicronemia
Viltolarsen (Viltepso)	analogous to Eleplinsen (Exondlys 51) but instead skipping exon 53	ASO-SSO/21 mer DNA PMO	2020.08	Duchenne Muscular Dystrophy
Casimersen (Amondys 45)	analogous to Eleplinsen (Exondlys 51) but instead skipping exon 45	ASO-SSO/22 mer DNA PMO	2021.02	Duchenne Muscular Dystrophy

*Phosphorodiamidate morpholino oligomers (PMO)
**Central nervous system
***Phosphorothioate
****Splice-switching oligonucleotide

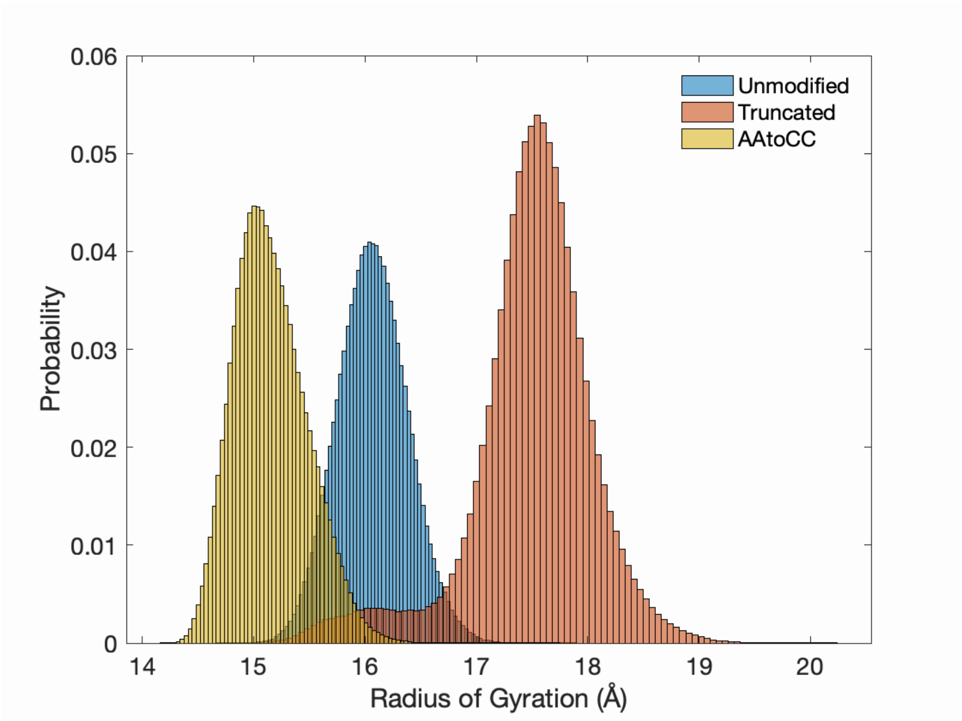

Figure S1: Radius of Gyration histogram.

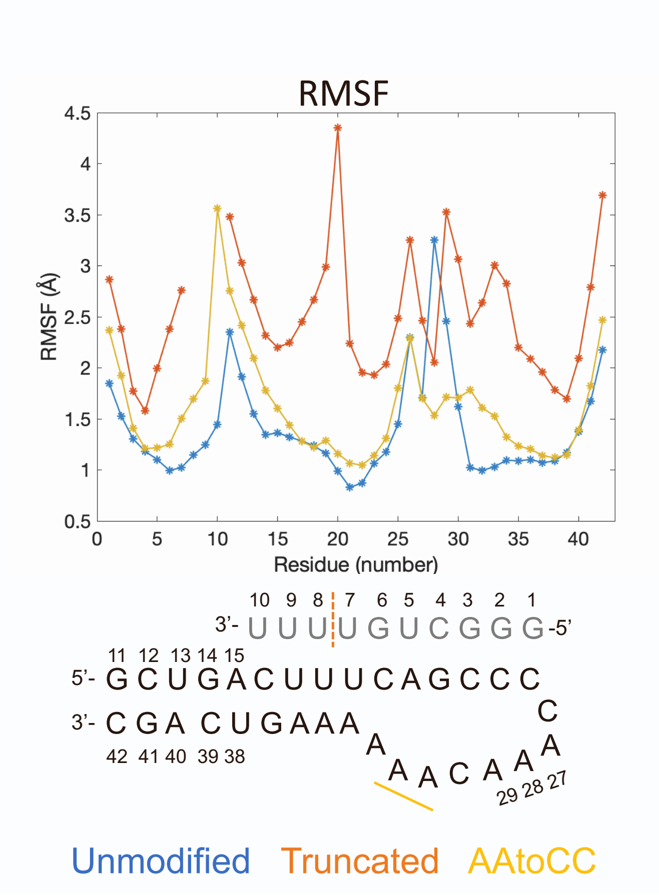

Figure S2: Per residue RMSF for the simulation window 100-2000ns. In the schematic figure of the ASO-hairpin, the truncation is marked with the orange dashed line and mutation is marked with yellow bold line.

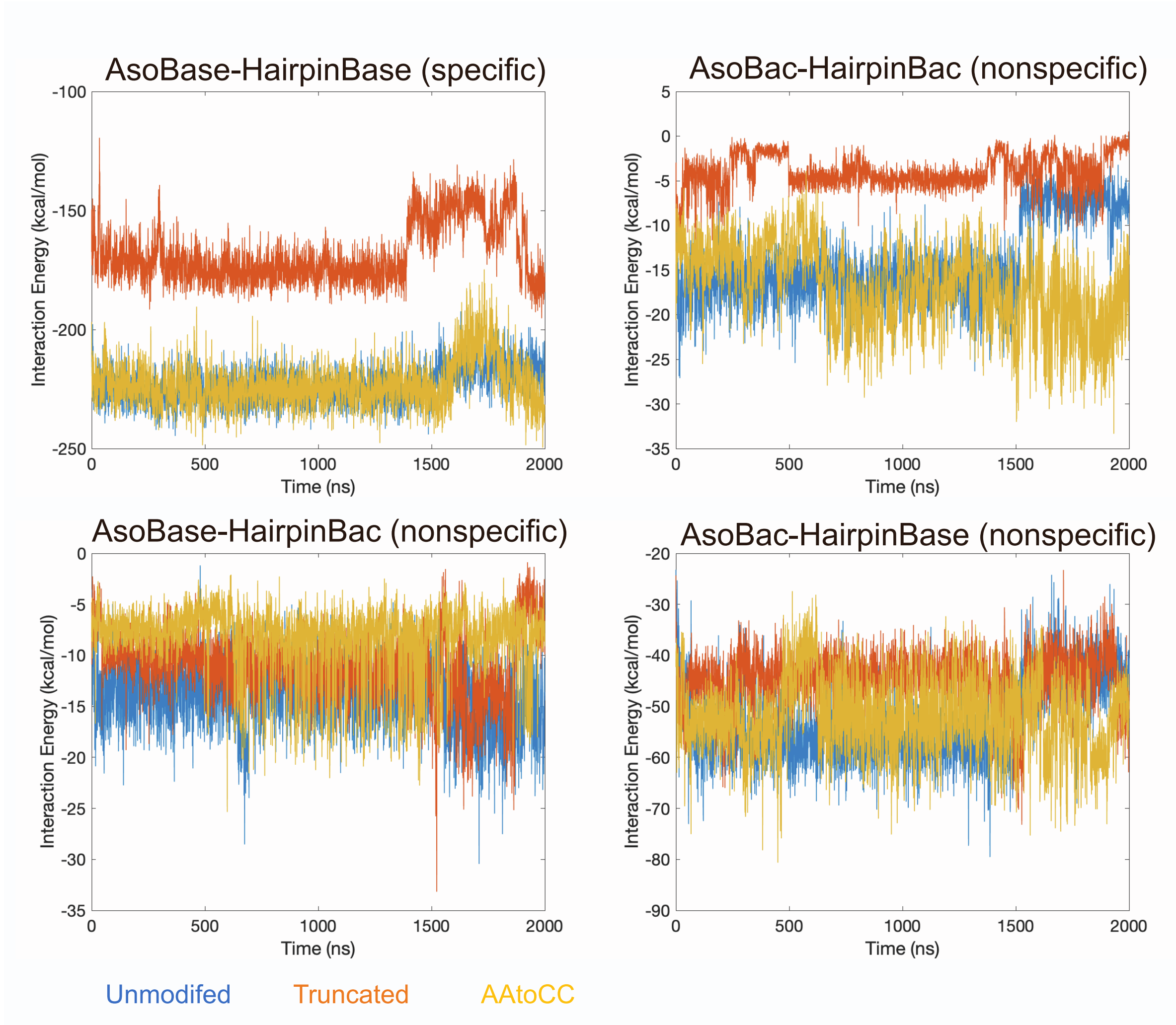

Figure S3: GROMACS interaction energies (sum of short-range electrostatic “Coulombic” and vdW “Lennard-Jones” interactions) for different atom groups of ASO-hairpin complex. Base constitutes atoms of the nucleobases and Bac constitutes atoms of the backbone. All interactions involving backbone atoms are referred to as nonspecific interactions.

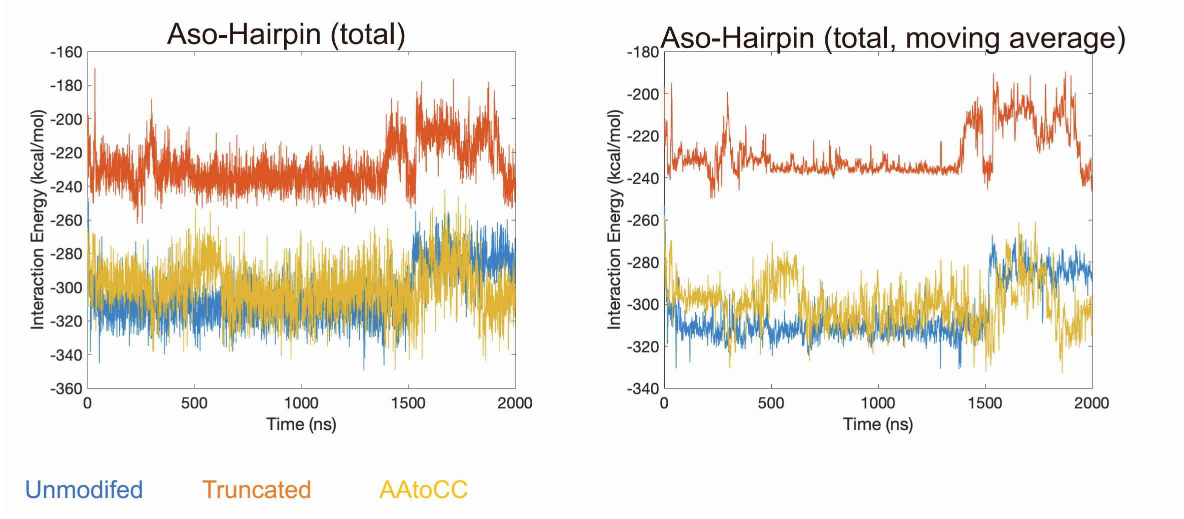

Figure S4: GROMACS total interaction energies.

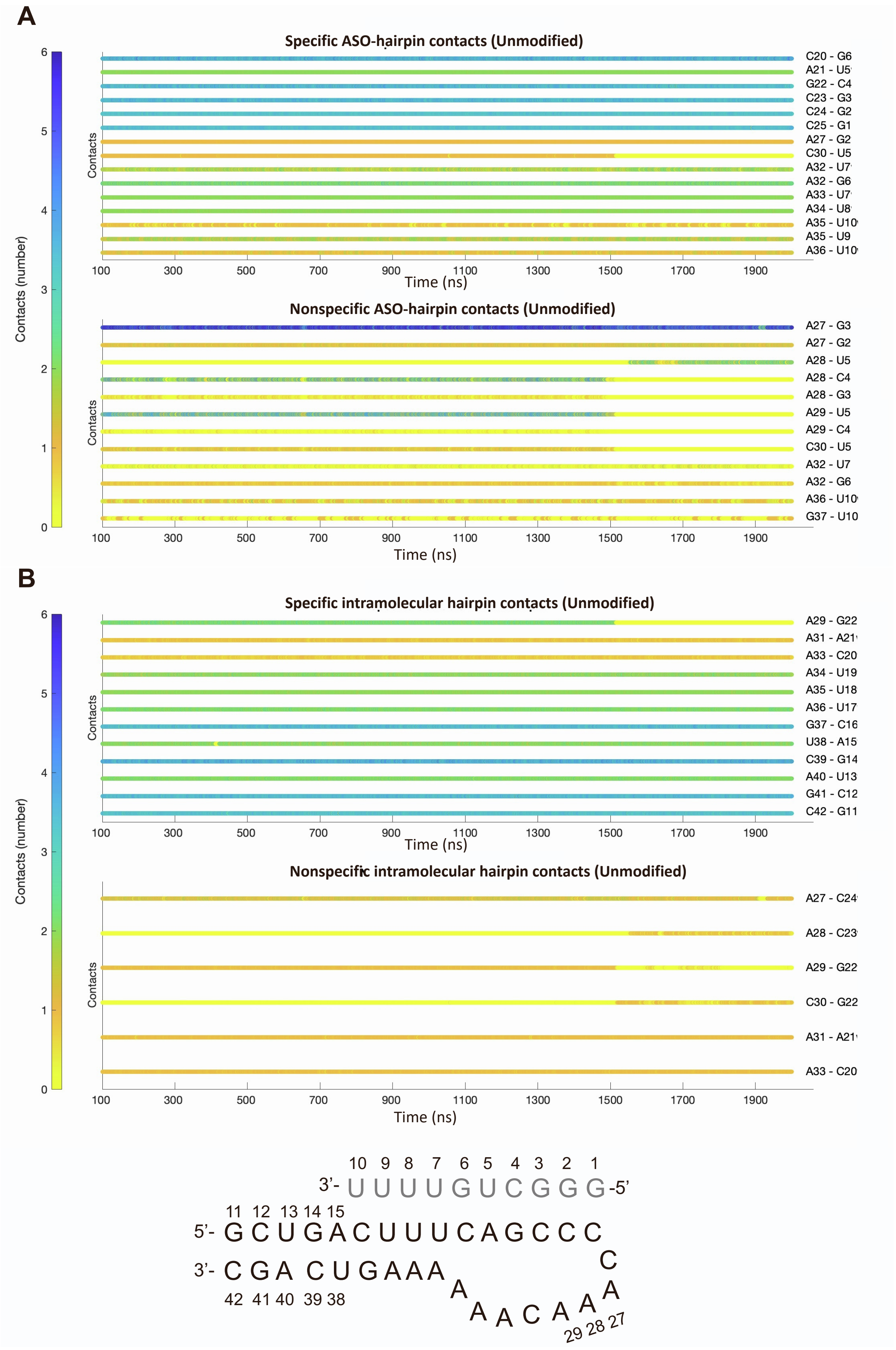

Figure S5: Dynamic contacts map for specific and nonspecific interactions: **A.** ASO-hairpin contacts and **B.** intramolecular hairpin contacts for the unmodified system.

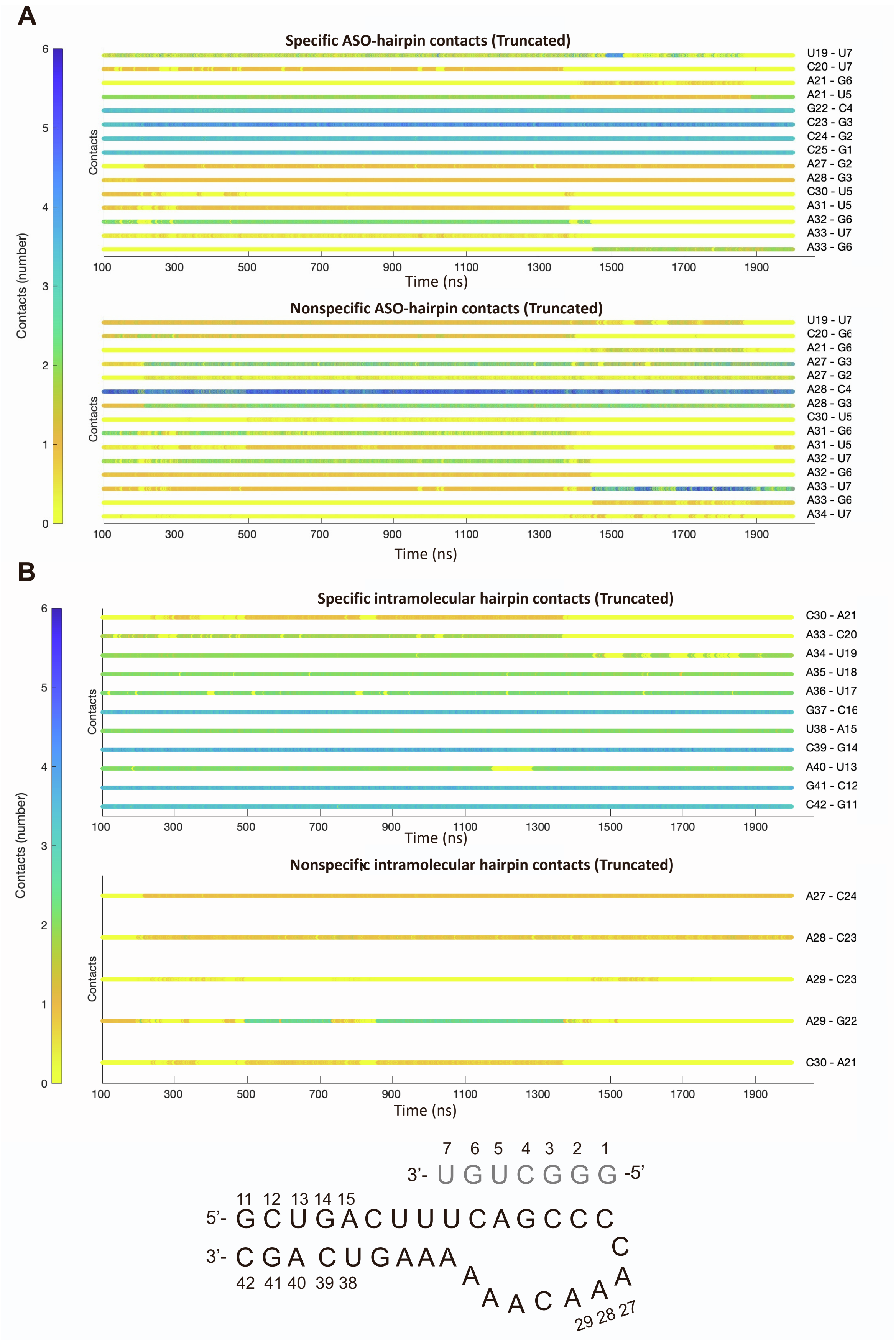

Figure S6: Dynamic contacts map for specific and nonspecific interactions: **A.** ASO-hairpin contacts and **B.** intramolecular hairpin contacts for the truncated ASO system.

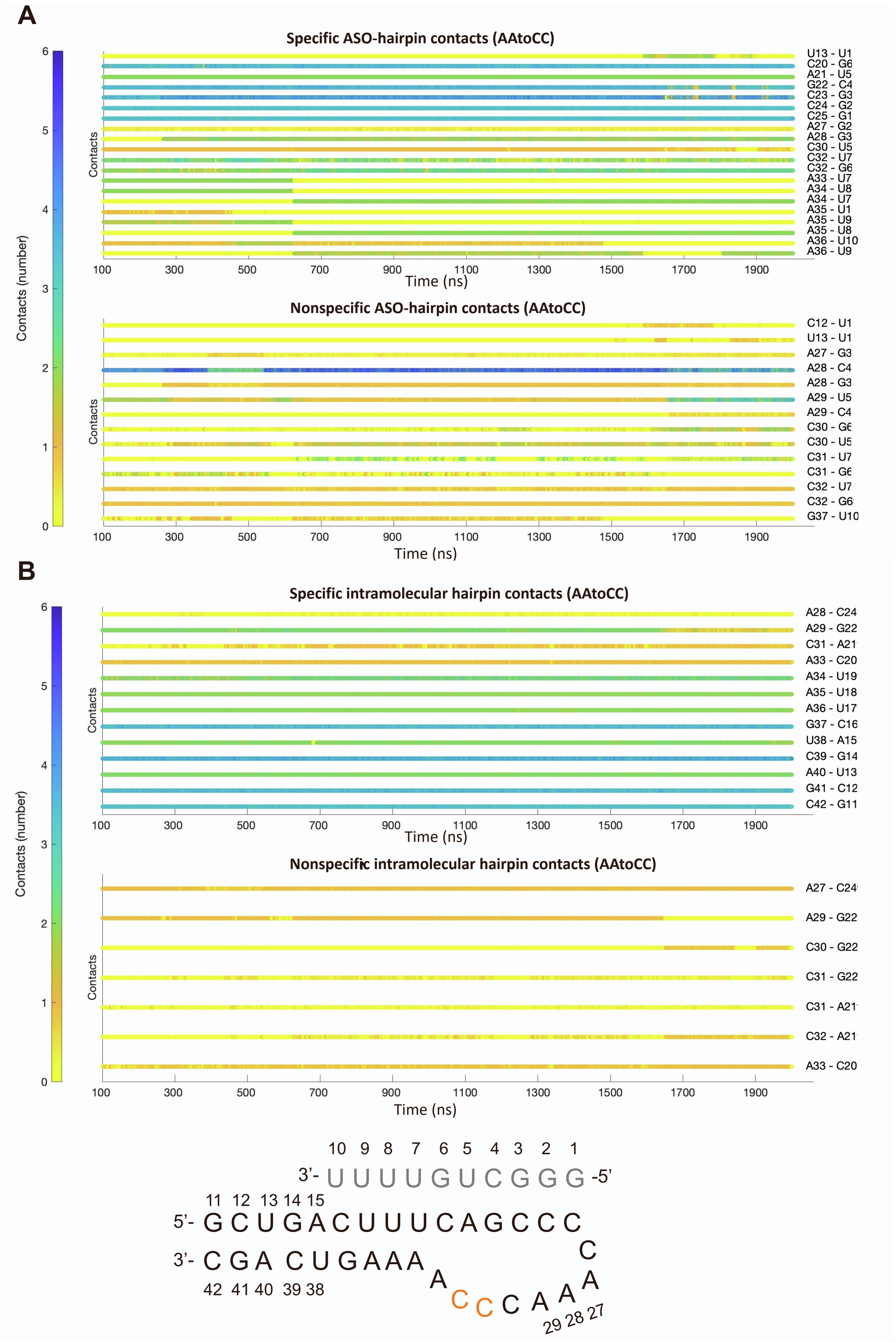

Figure S7: Dynamic contacts map for specific and nonspecific interactions: **A.** ASO-hairpin contacts and **B.** intramolecular hairpin contacts for the AAtoCC system.

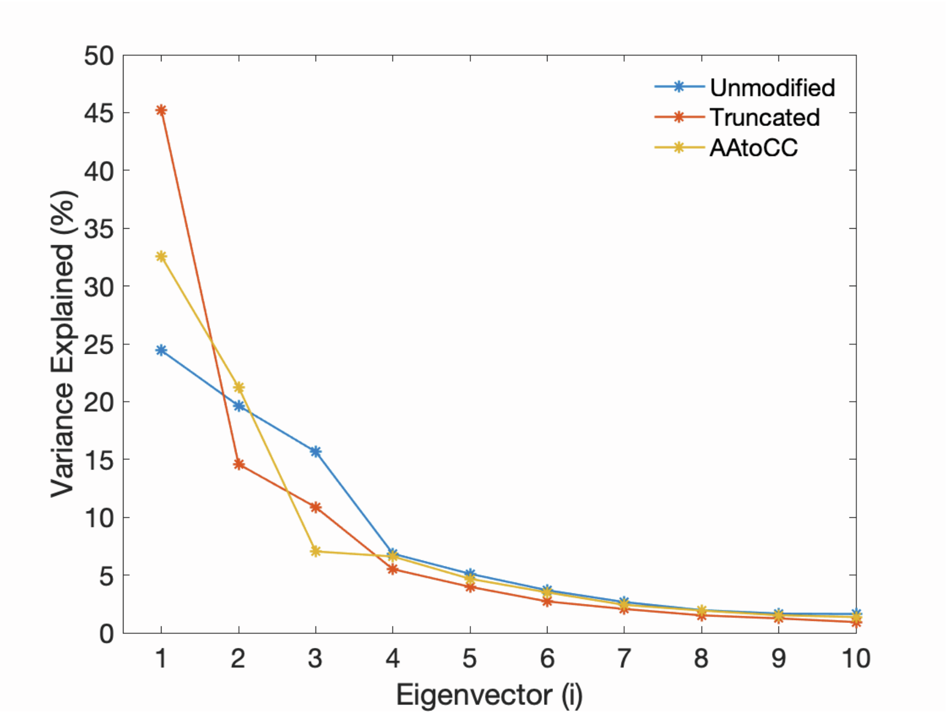

Figure S8: Principal components analysis, showing the variance explained for the first 10 eigenvectors.

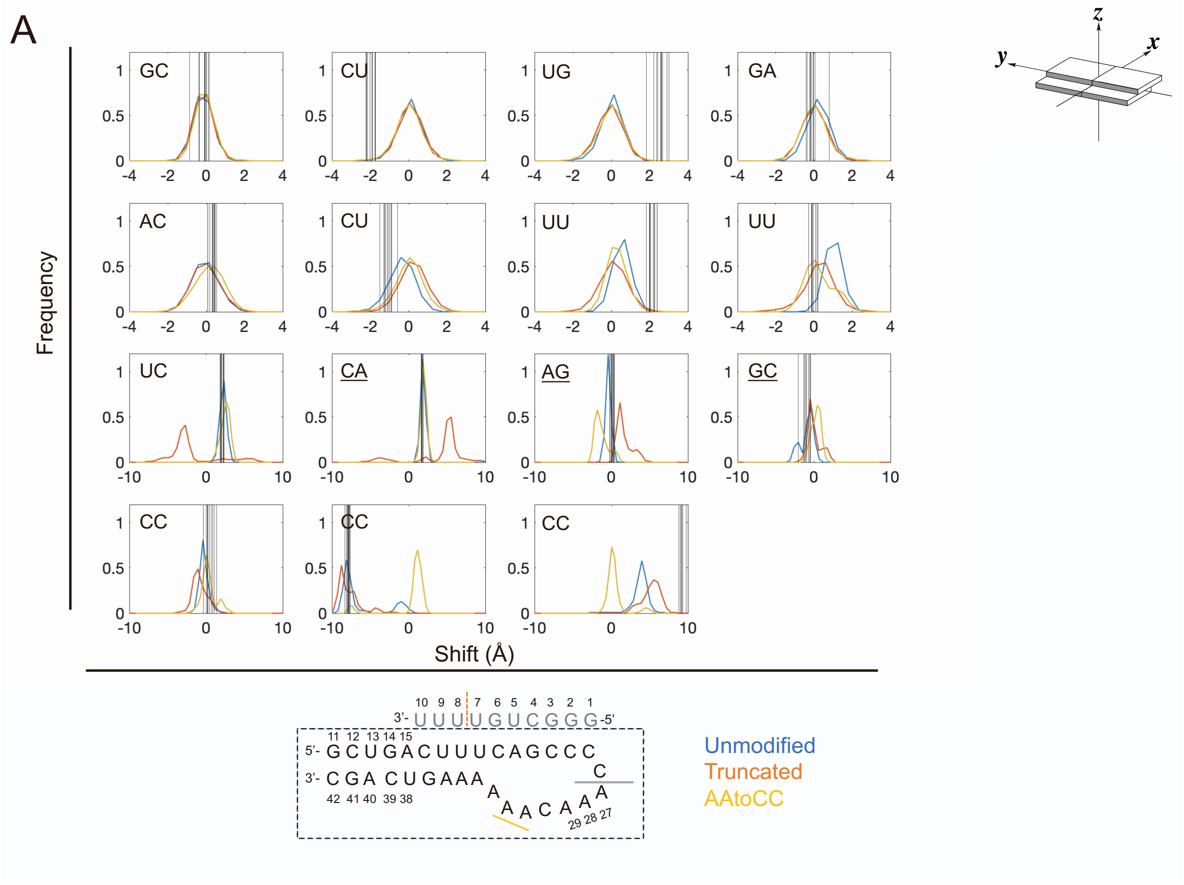

B

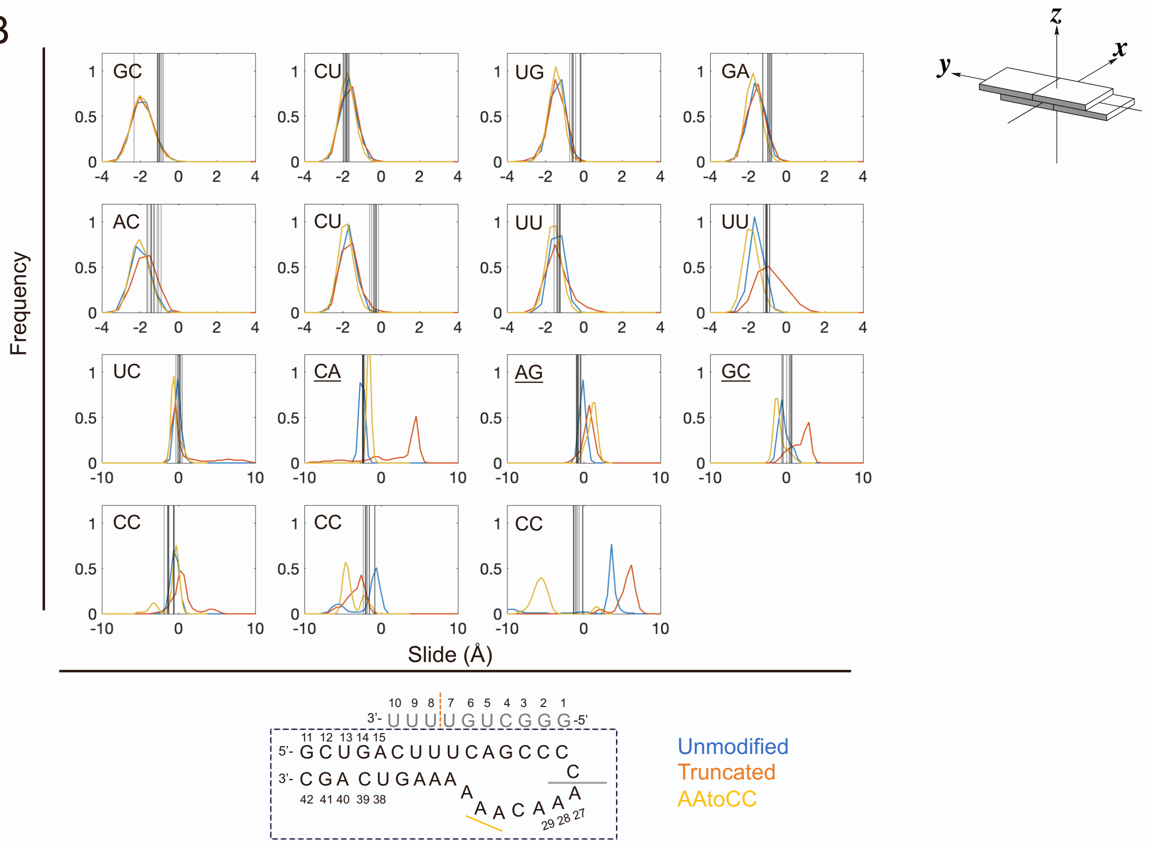

Frequency
GC
CU
UG
GA
AC
CU
UU
UU
UC
CA
AG
GC
CC
CC
CC
Slide (Å)
z
y
x
10 9 8 7 6 5 4 3 2 1
3'- U U U U G U C G G G -5'
11 12 13 14 15
5'- G C U G A C U U U C A G C C C
3'- C G A C U G A A A
42 41 40 39 38
A A A C A A A C
29 28 27
Unmodified
Truncated
AAtoCC

C

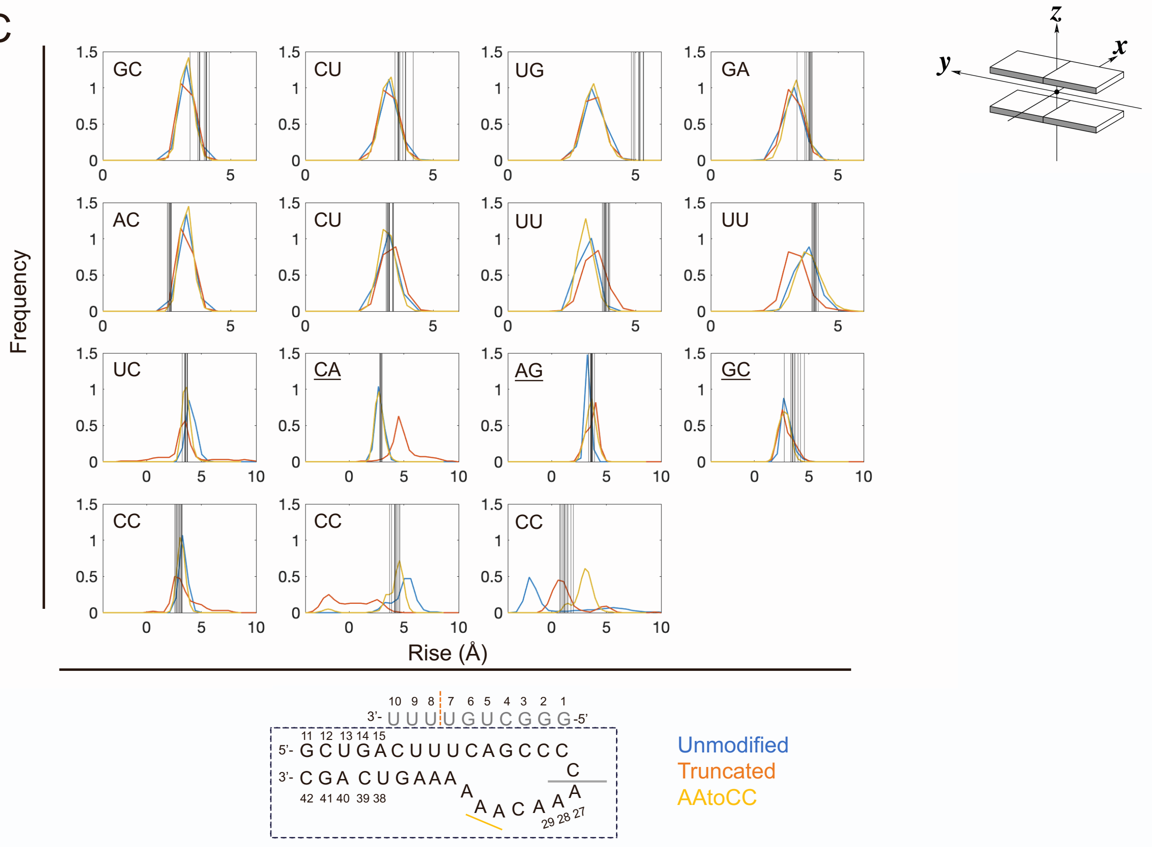

Frequency
GC
CU
UG
GA
AC
CU
UU
UU
UC
CA
AG
GC
CC
CC
CC
Rise (Å)
z
y
x
10 9 8 7 6 5 4 3 2 1
3'- U U U U G U C G G G -5'
11 12 13 14 15
5'- G C U G A C U U U C A G C C C
3'- C G A C U G A A A
42 41 40 39 38
A A A C A A A C
29 28 27
Unmodified
Truncated
AAtoCC

D

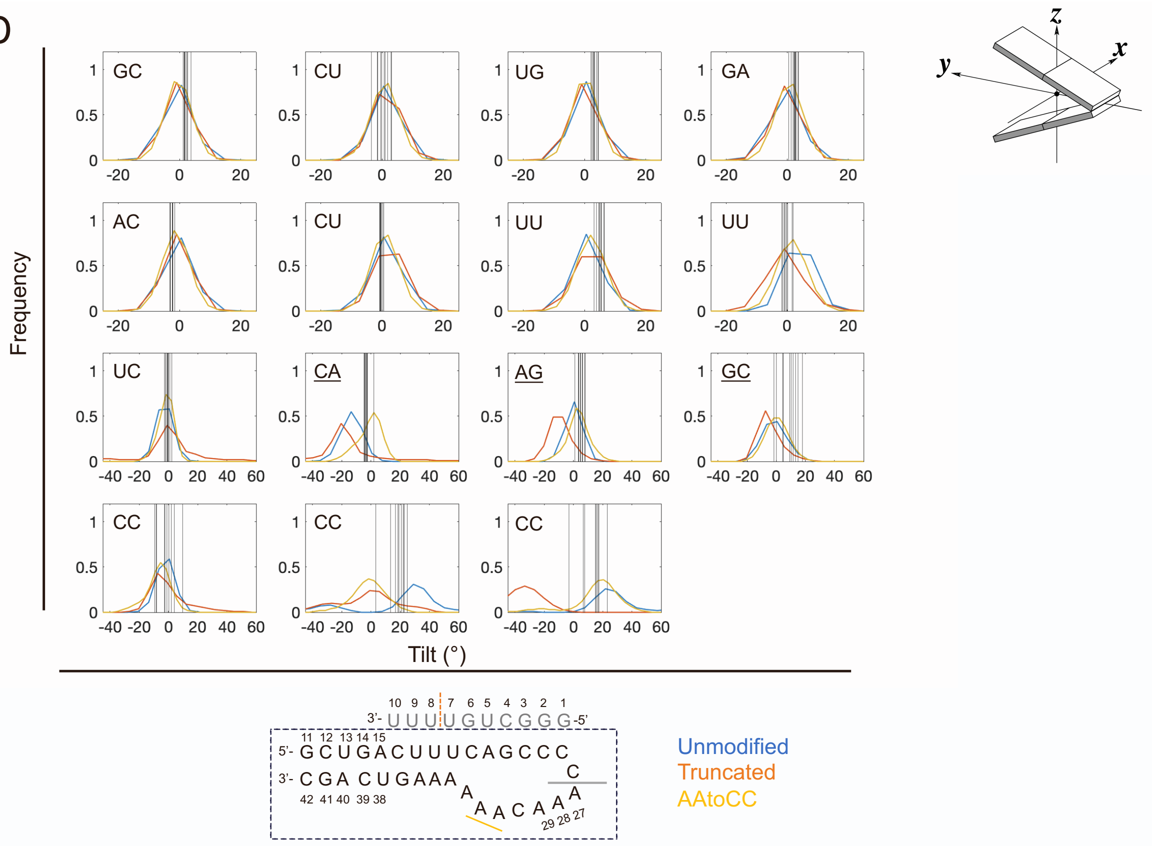
Frequency
Tilt (°)
GC
CU
UG
GA
AC
UU
UC
CA
AG
CC
z
x
y
3'- U U U U G U C G G G -5'
5'- G C U G A C U U U C A G C C C
3'- C G A C U G A A A
A A C A A A C
Unmodified
Truncated
AAtoCC

E

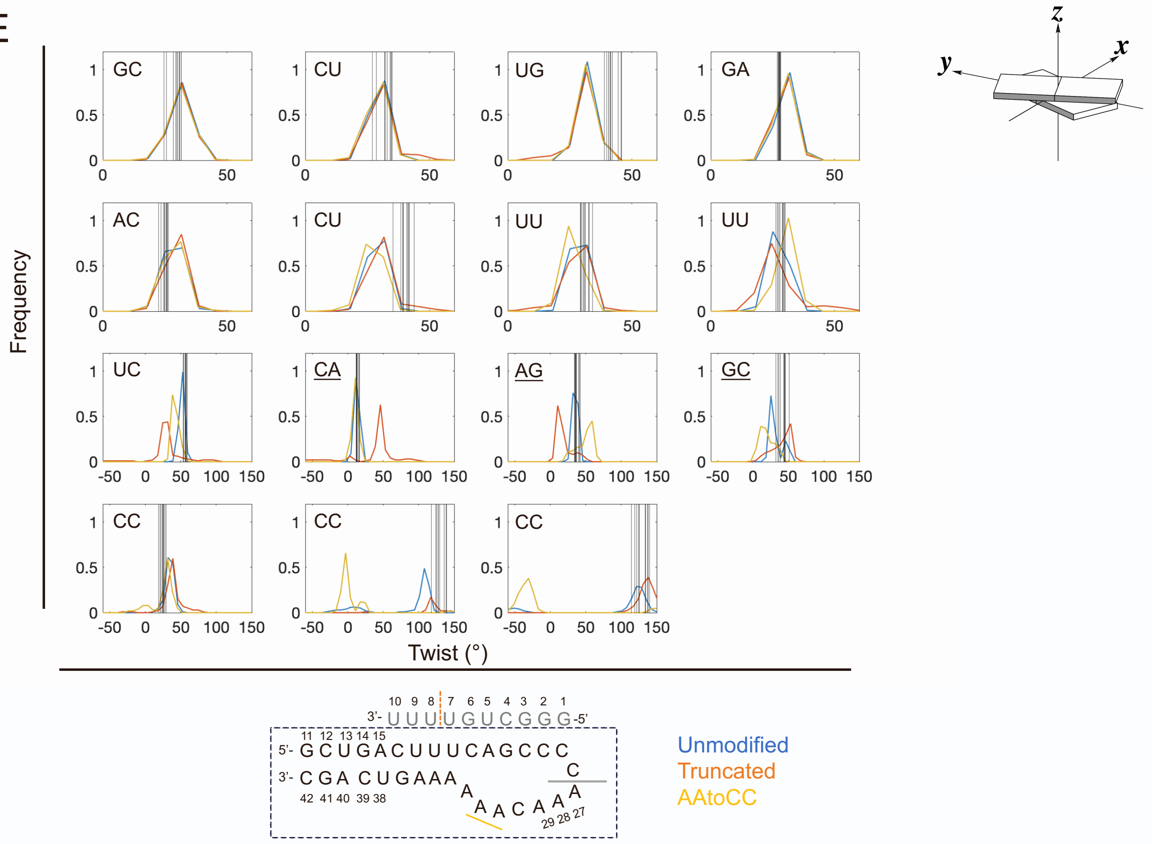
Frequency
Twist (°)
GC
CU
UG
GA
AC
UU
UC
CA
AG
CC
z
x
y
3'- U U U U G U C G G G -5'
5'- G C U G A C U U U C A G C C C
3'- C G A C U G A A A
A A C A A A C
Unmodified
Truncated
AAtoCC

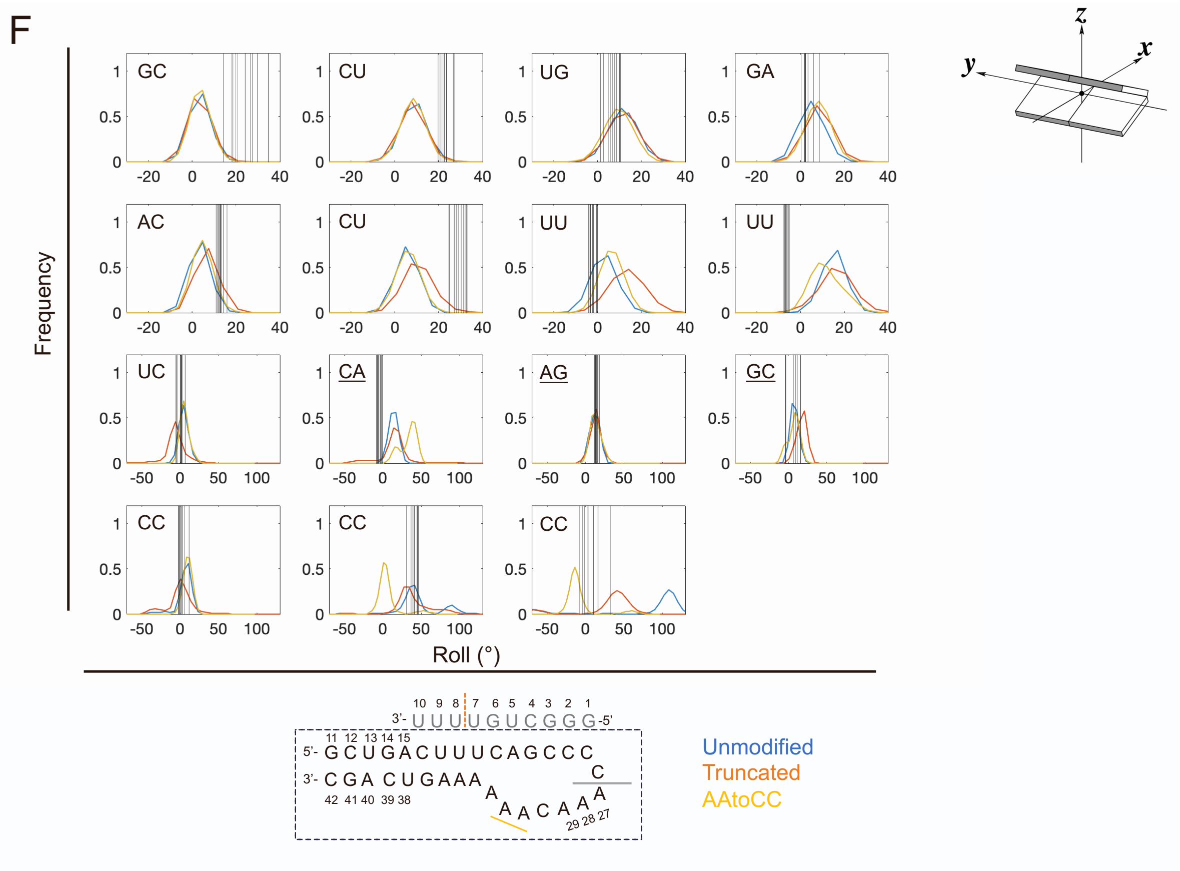

Figure S9: Value distributions of base pair step parameters for the hairpin formed between bases 11-26 (denoted as “strand 1”) and bases 27-42 (“strand 2”). The b.p. step for strand 1 is shown as labels in each plot, where the b.p. steps involved in interactions with the mutated A31A32 bases for the mutated AAtoCC variant are underlined. Vertical black lines constitute the corresponding values for the NMR models 1-10 (PDB ID: 1YMO). **A.** Shift **B.** Slide **C.** Rise **D**. Tilt **E.** Twist **F.** Roll.

A

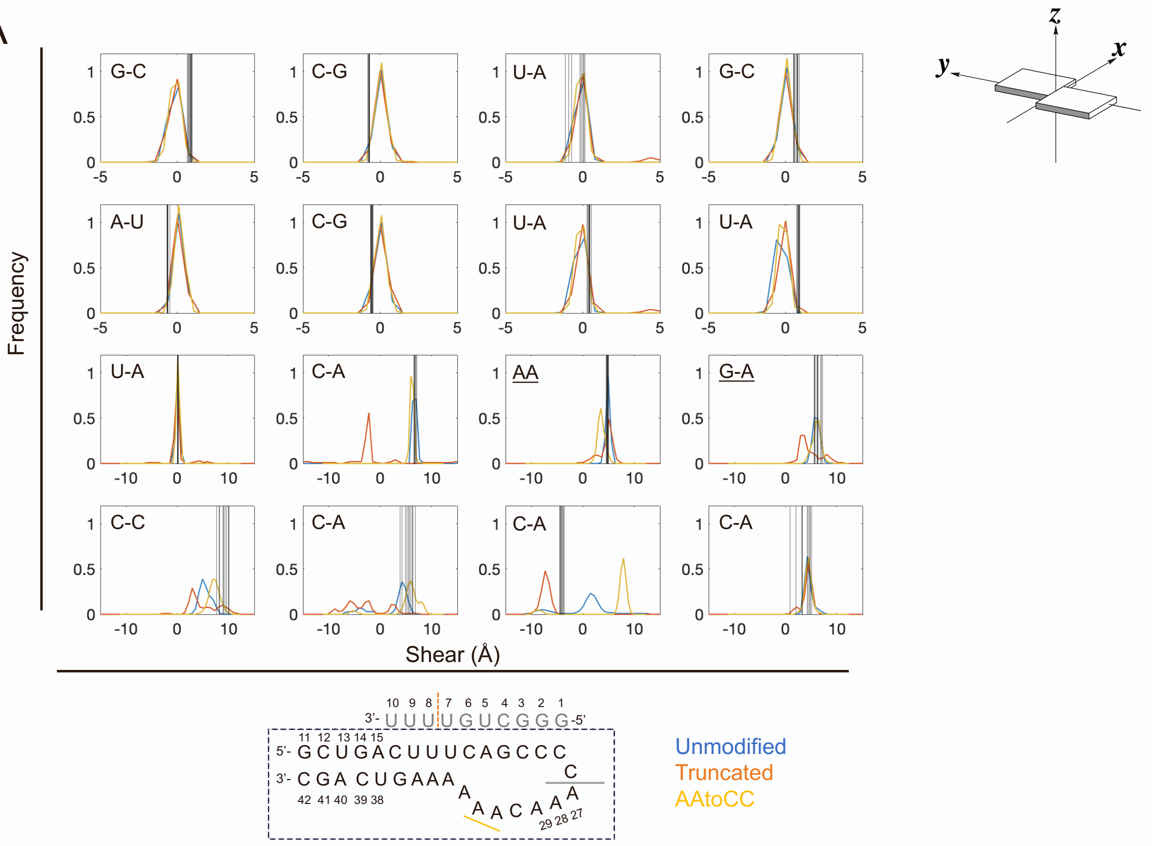
Frequency
G-C
C-G
U-A
G-C
A-U
C-G
U-A
U-A
U-A
C-A
AA
G-A
C-C
C-A
C-A
C-A
Shear (Å)
Unmodified
Truncated
AAtoCC
z
x
y

B

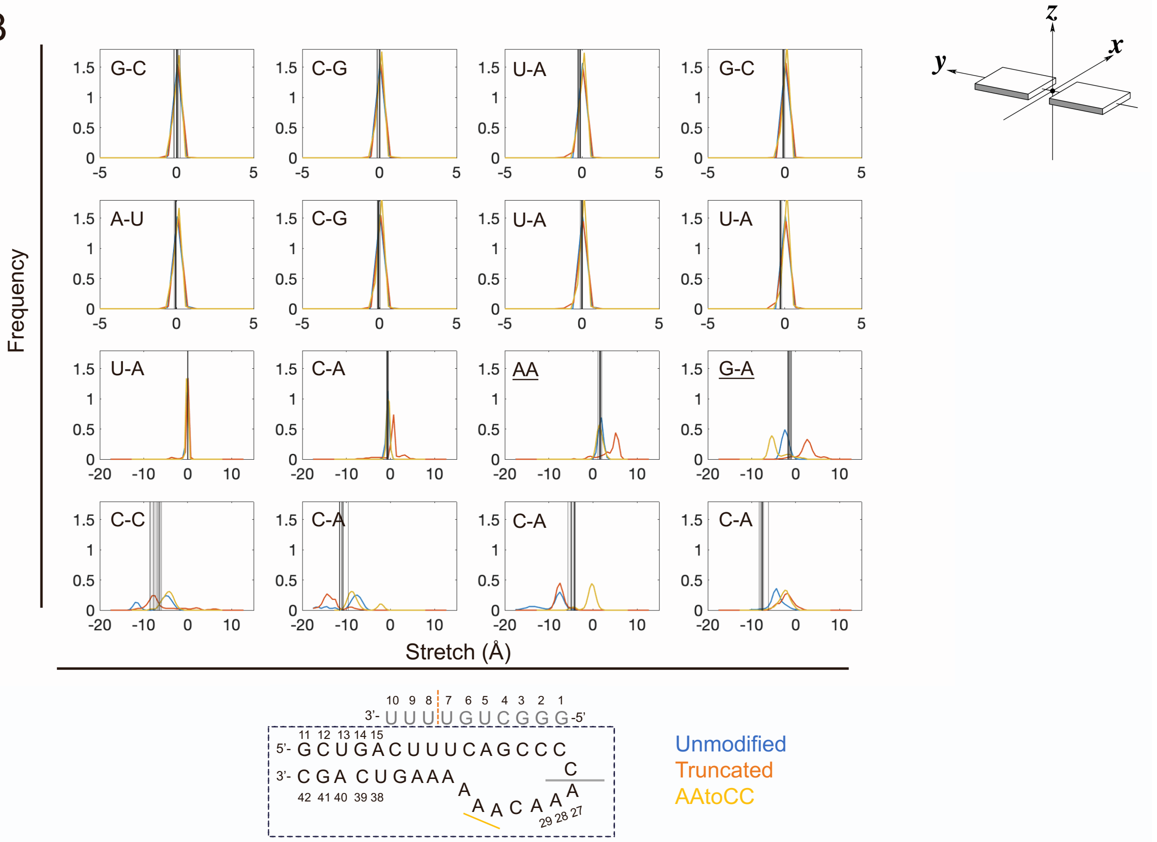
Frequency
G-C
C-G
U-A
G-C
A-U
C-G
U-A
U-A
U-A
C-A
AA
G-A
C-C
C-A
C-A
C-A
Stretch (Å)
Unmodified
Truncated
AAtoCC
z
x
y

C

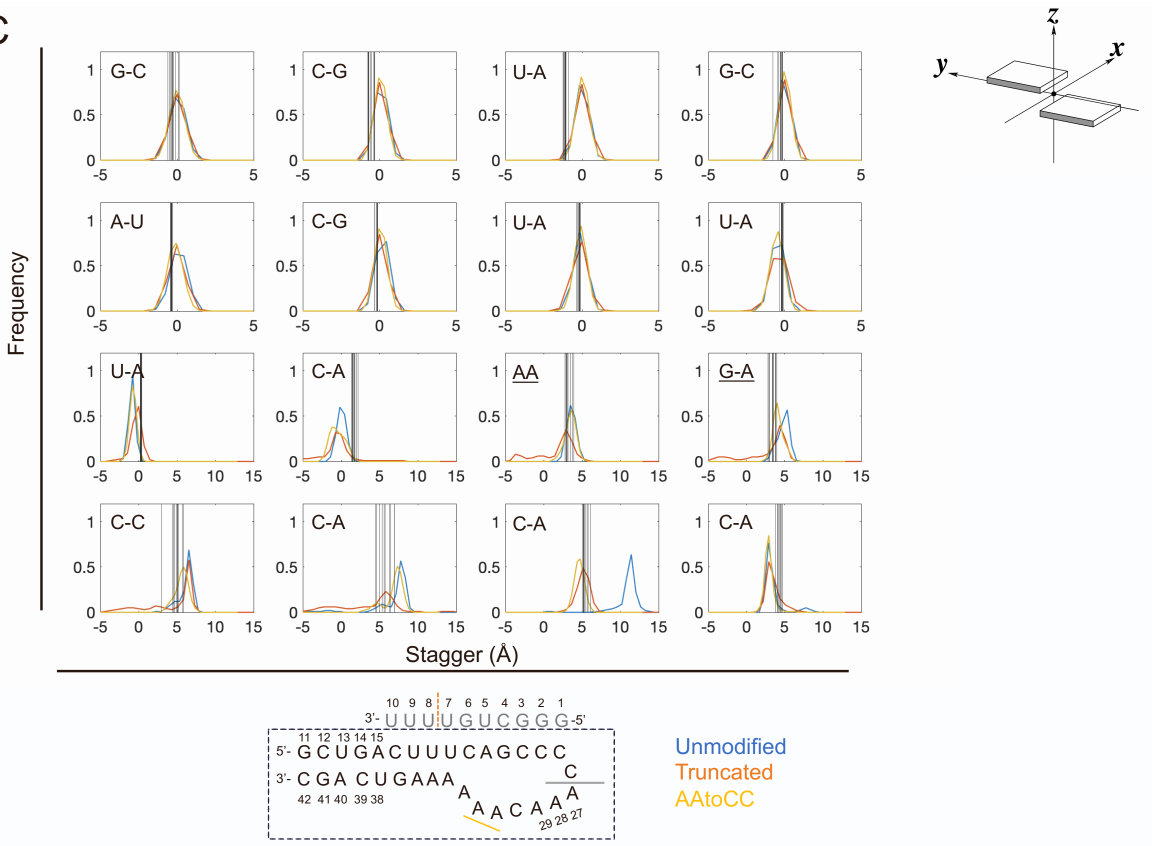
Frequency
G-C
C-G
U-A
G-C
A-U
C-G
U-A
U-A
U-A
C-A
AA
G-A
C-C
C-A
C-A
C-A
Stagger (Å)
z
y
x
10 9 8 7 6 5 4 3 2 1
3'- U U U U G U C G G G -5'
11 12 13 14 15
5'- G C U G A C U U U C A G C C C
3'- C G A C U G A A A
A A C A A A C
42 41 40 39 38
29 28 27
Unmodified
Truncated
AAtoCC

D

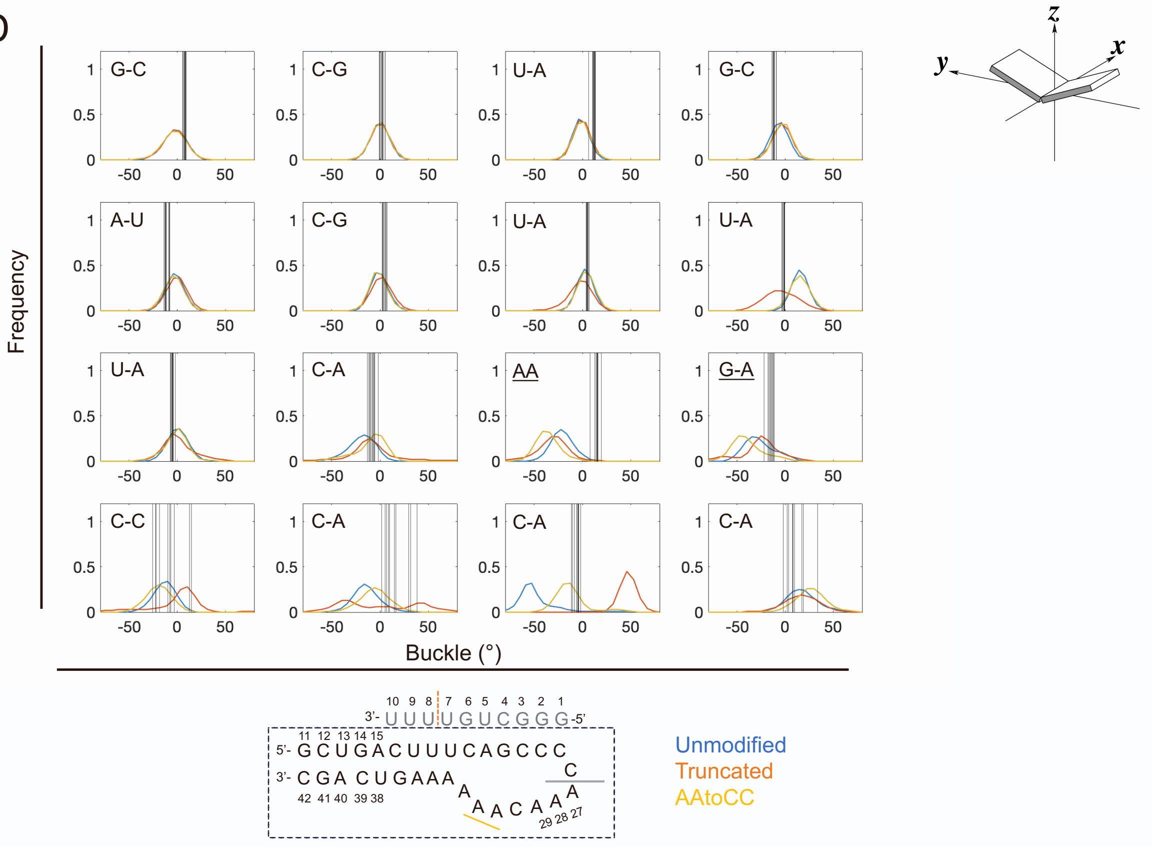
Frequency
G-C
C-G
U-A
G-C
A-U
C-G
U-A
U-A
U-A
C-A
AA
G-A
C-C
C-A
C-A
C-A
Buckle (°)
z
y
x
10 9 8 7 6 5 4 3 2 1
3'- U U U U G U C G G G -5'
11 12 13 14 15
5'- G C U G A C U U U C A G C C C
3'- C G A C U G A A A
A A C A A A C
42 41 40 39 38
29 28 27
Unmodified
Truncated
AAtoCC

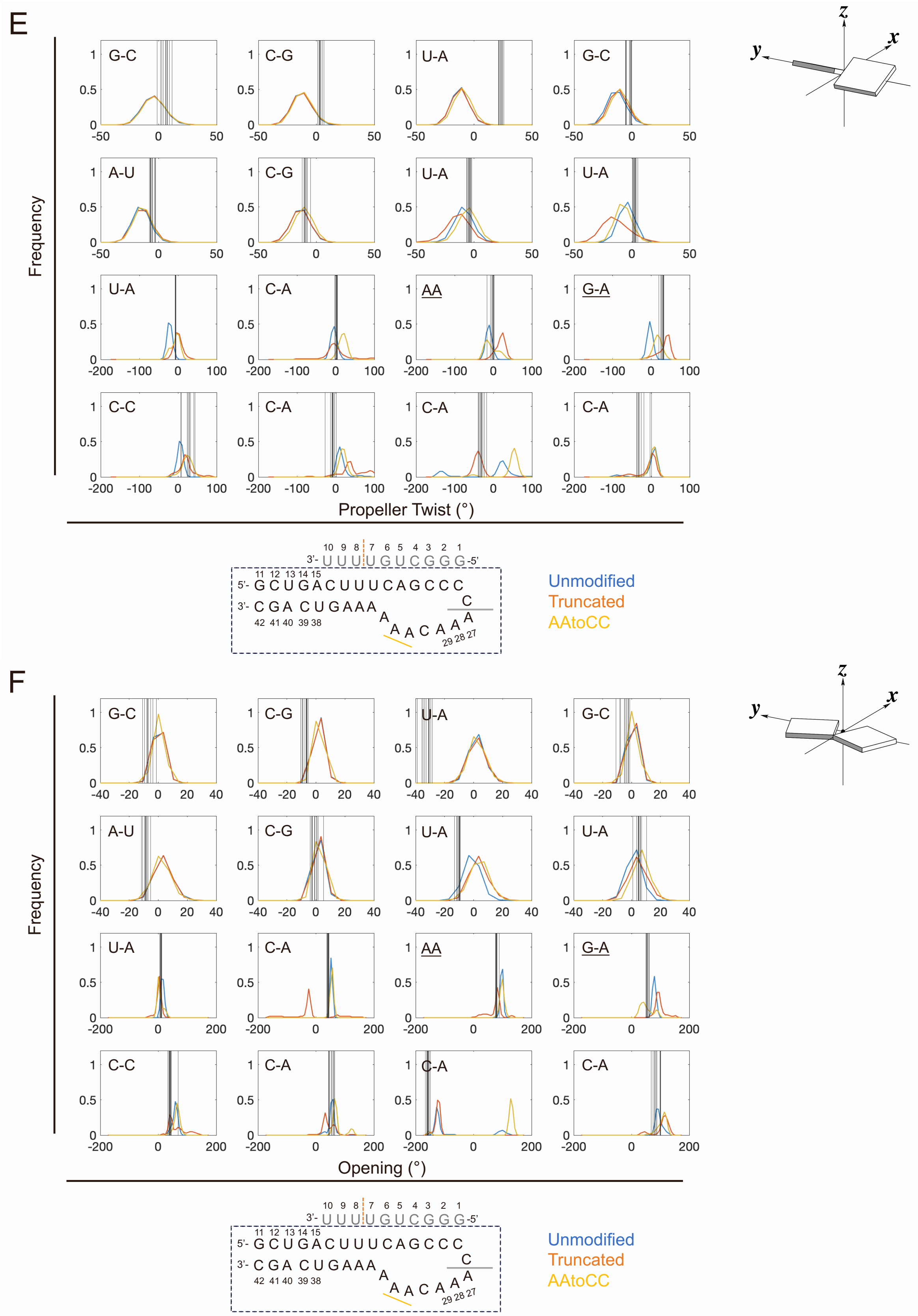

Figure S10: Value distributions of base pair parameters for the hairpin formed between bases 11-26 (denoted as "strand 1") and bases 27-42 ("strand 2"). The b.p. between strand 1 and strand 2 are shown as labels in each plot, where the b.p. steps involved in interactions with the mutated A31A32 bases for the mutated AAtoCC variant are underlined. Vertical black lines constitute the corresponding values for the NMR models 1-10 (PDB id: 1YMO). **A.** Shear **B.** Stretch **C.** Stagger **D**. Buckle **E.** Propeller twist **F.** Opening.

A

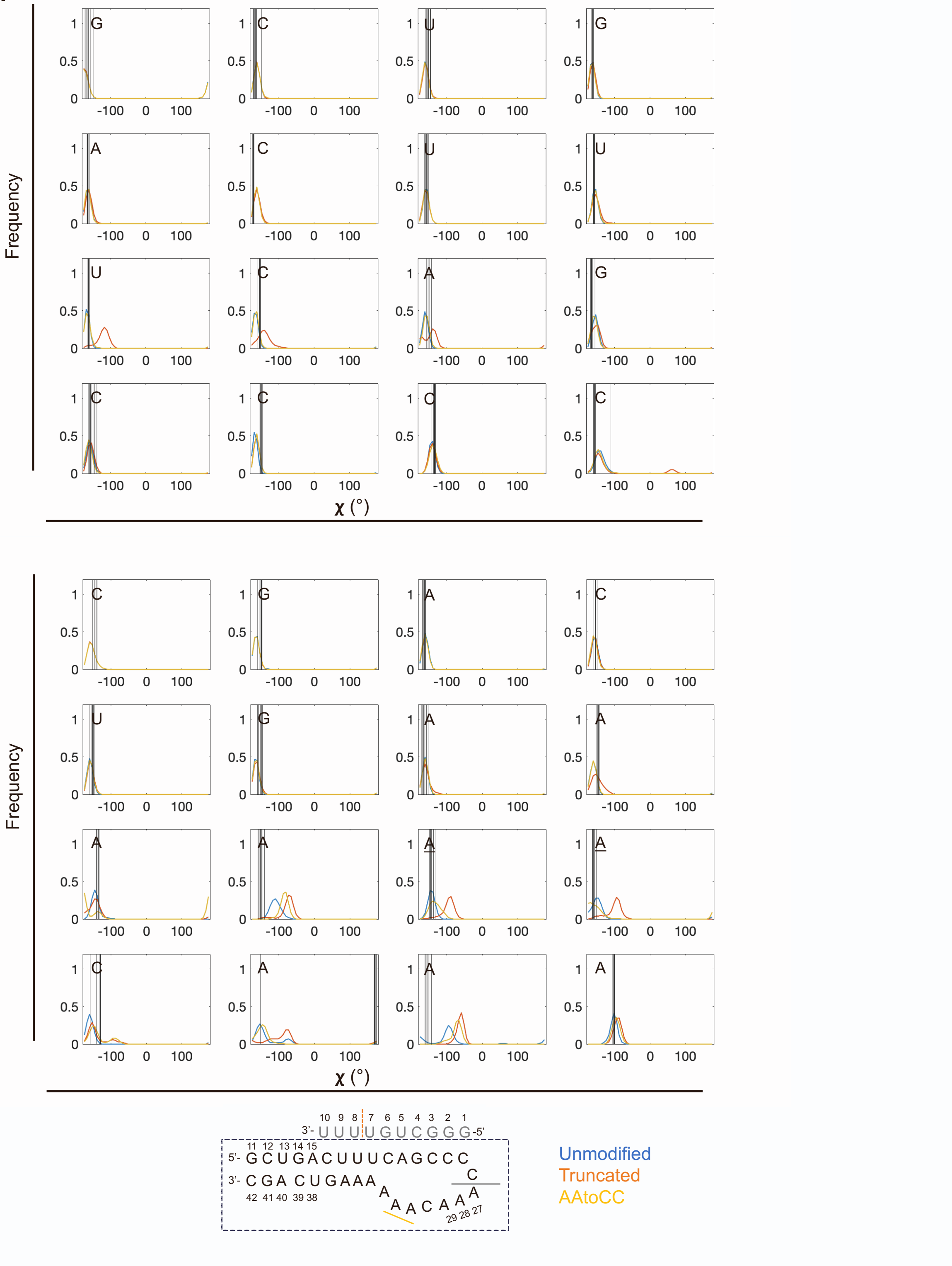
Frequency
χ (°)
Unmodified
Truncated
AAtoCC
3'- U U U U G U C G G G -5'
5'- G C U G A C U U U C A G C C C
3'- C G A C U G A A A A A A C A A A C

B

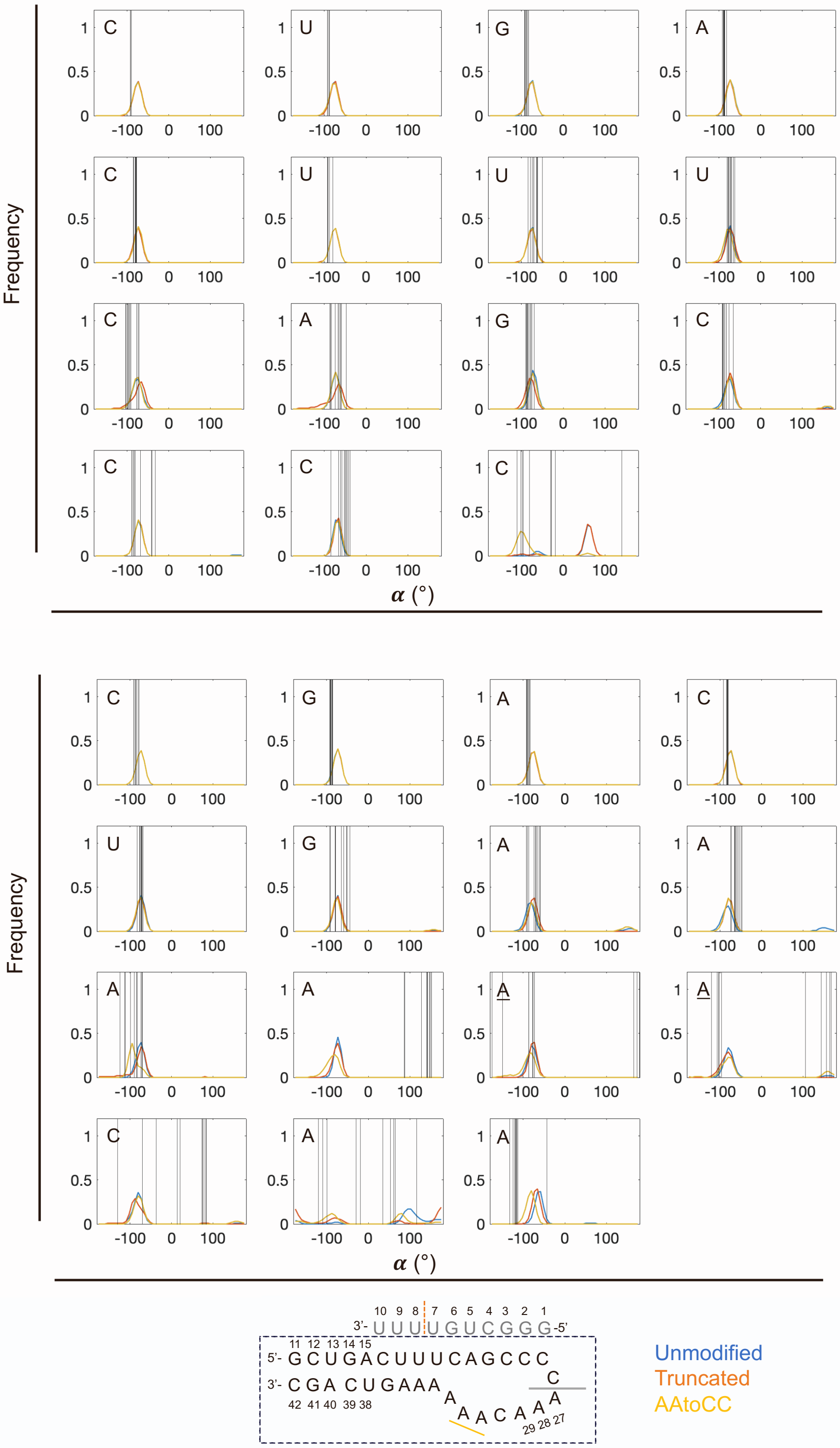
Frequency
α (°)
10 9 8 7 6 5 4 3 2 1
3'- U U U U G U C G G G -5'
11 12 13 14 15
5'- G C U G A C U U U C A G C C C
3'- C G A C U G A A A A A C A A A C
42 41 40 39 38
29 28 27
Unmodified
Truncated
AAtoCC

C

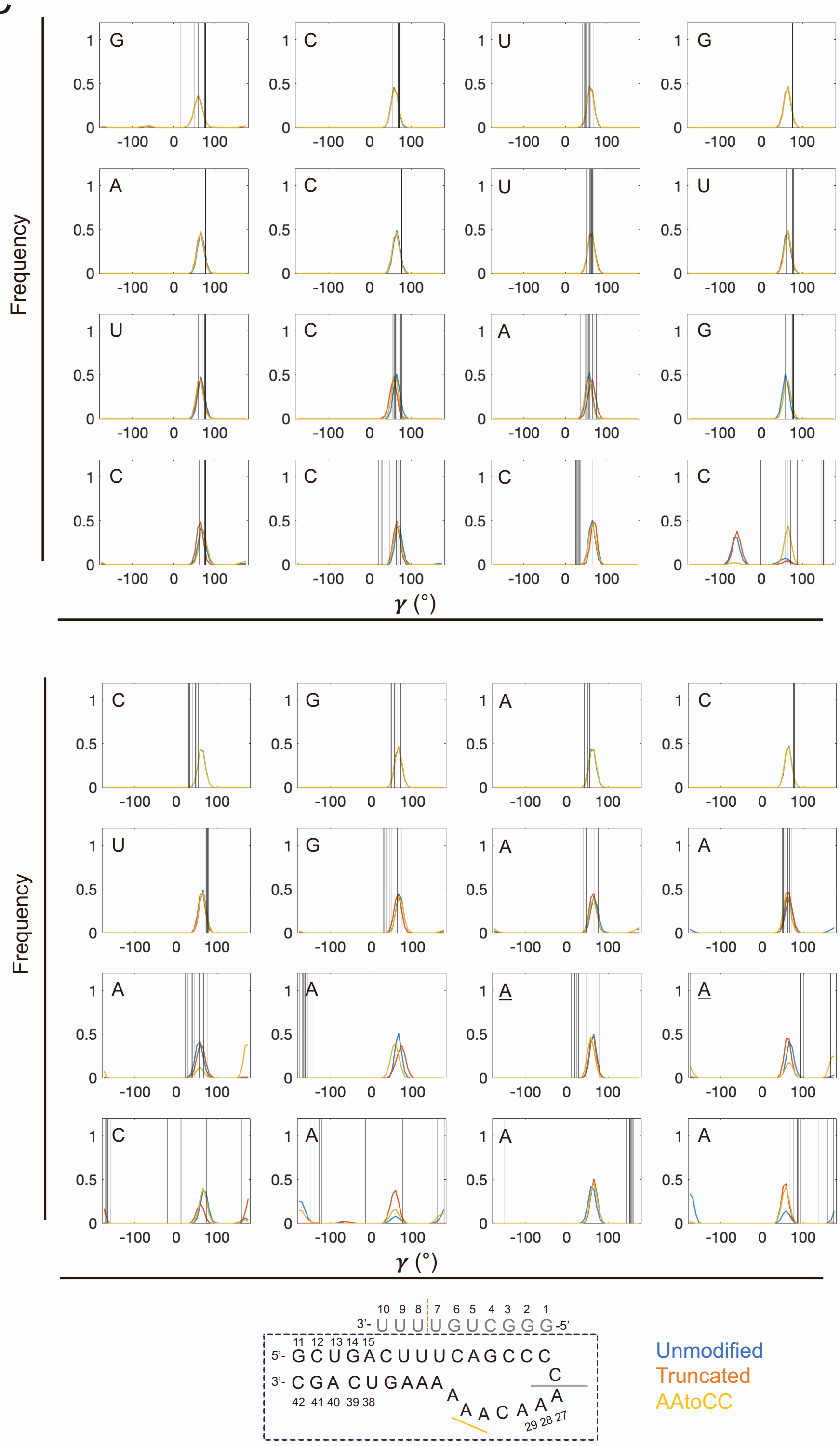
Frequency
γ (°)
3'- U U U U G U C G G G -5'
5'- G C U G A C U U U C A G C C C
3'- C G A C U G A A A
Unmodified
Truncated
AAtoCC

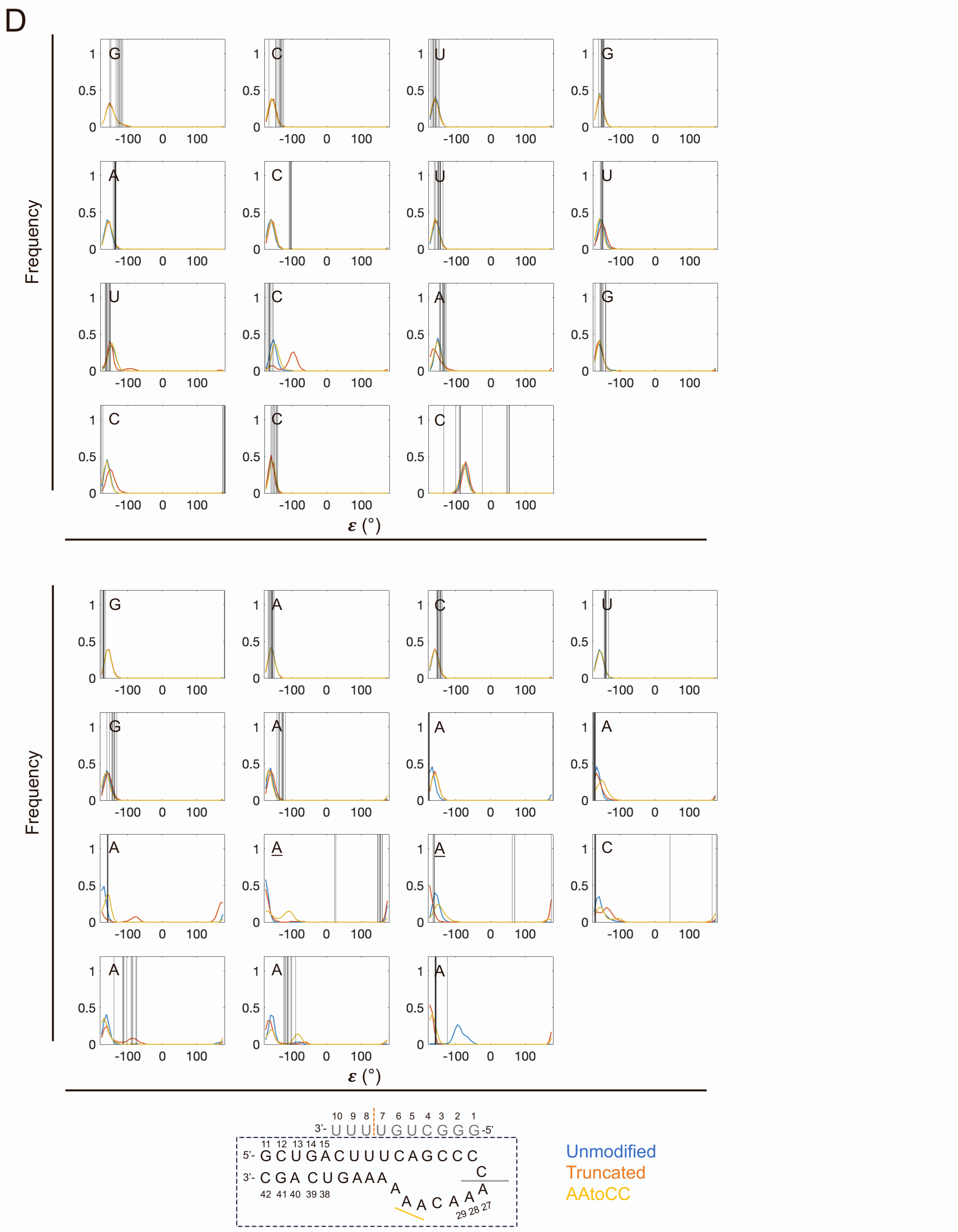

D
Frequency
ε (°)
Unmodified
Truncated
AAtoCC

E

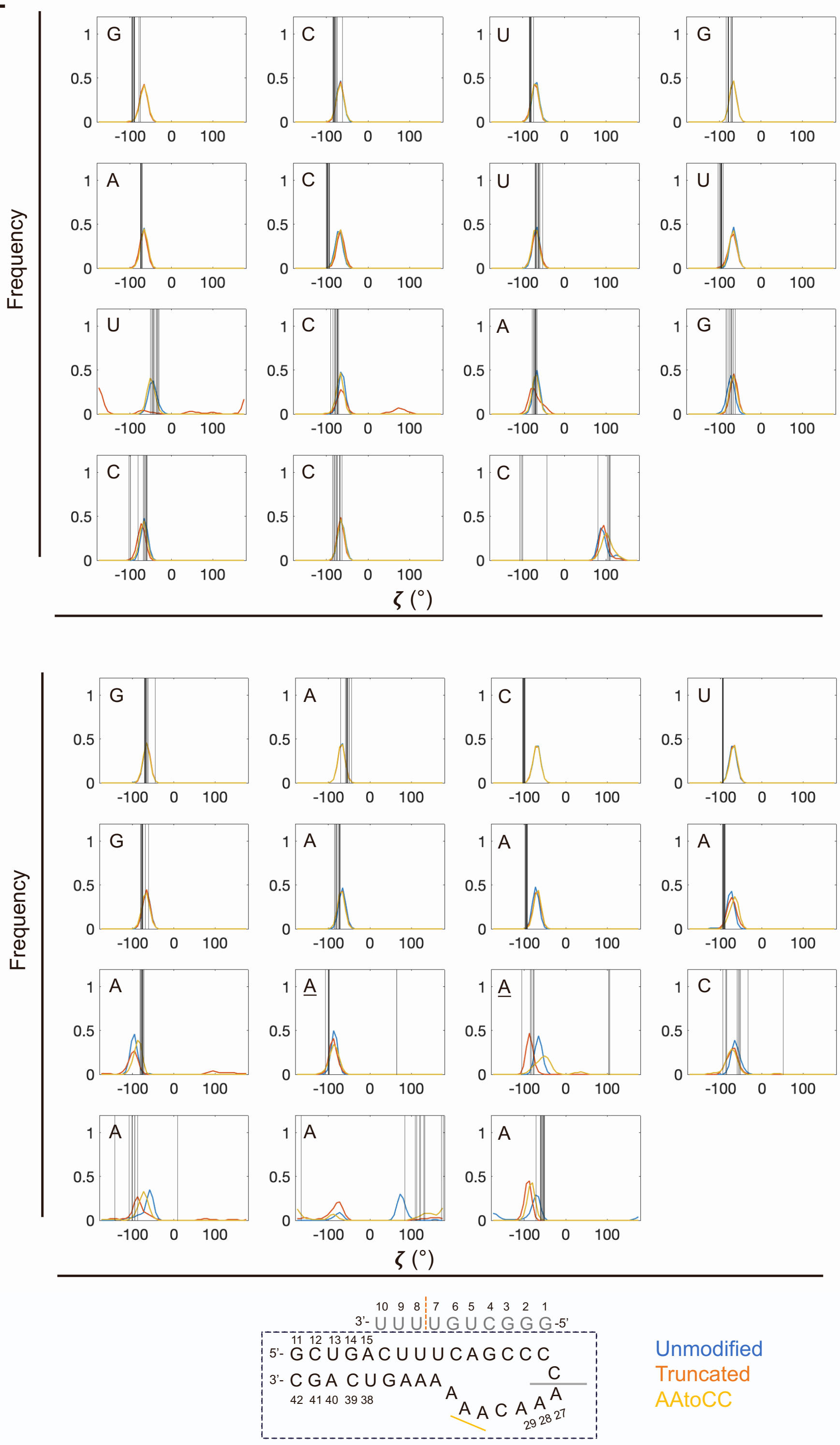

Figure S11: Value distributions of torsional angels for the hairpin formed between bases 11-26 (denoted as "strand 1") and bases 27-42 ("strand 2"). The upper panels constitute torsional angels for strand 1, shown in the 5'-3' direction, and the lower panels constitute torsional angels for strand 2, shown in the 3'-5' direction. The A31A32 bases, which are mutated in the mutated AAtoCC variant are underlined. Vertical black lines constitute the corresponding values for the NMR models 1-10 (PDB id: 1YMO). **A.** χ **B.** α **C.** γ **D.** ε **E.** ζ.

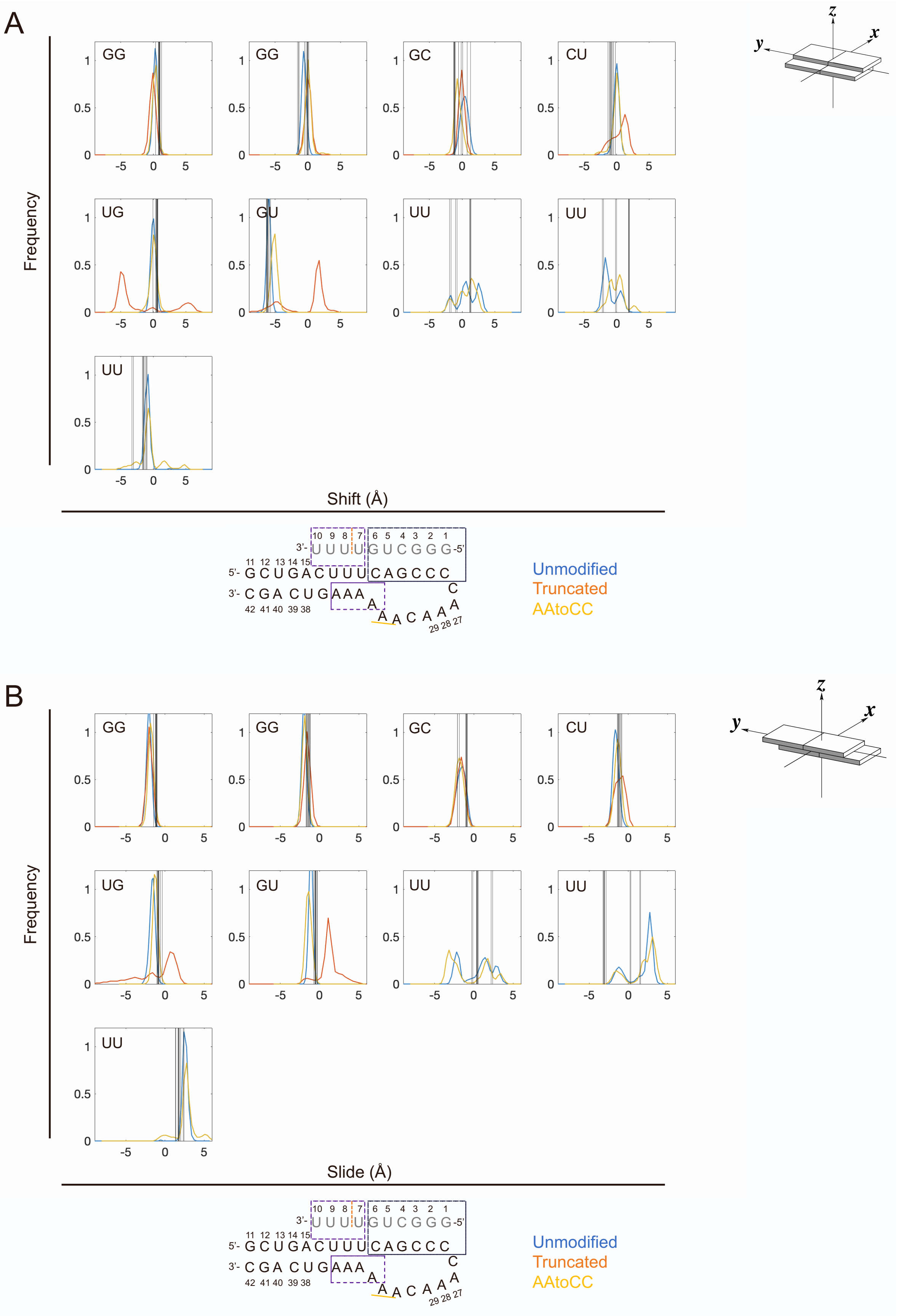
A
B
GG
GC
CU
UG
GU
UU
Frequency
Shift (Å)
Slide (Å)
z
y
x
3'- U U U U G U C G G G -5'
5'- G C U G A C U U U C A G C C C
3'- C G A C U G A A A
Unmodified
Truncated
AAtoCC

C

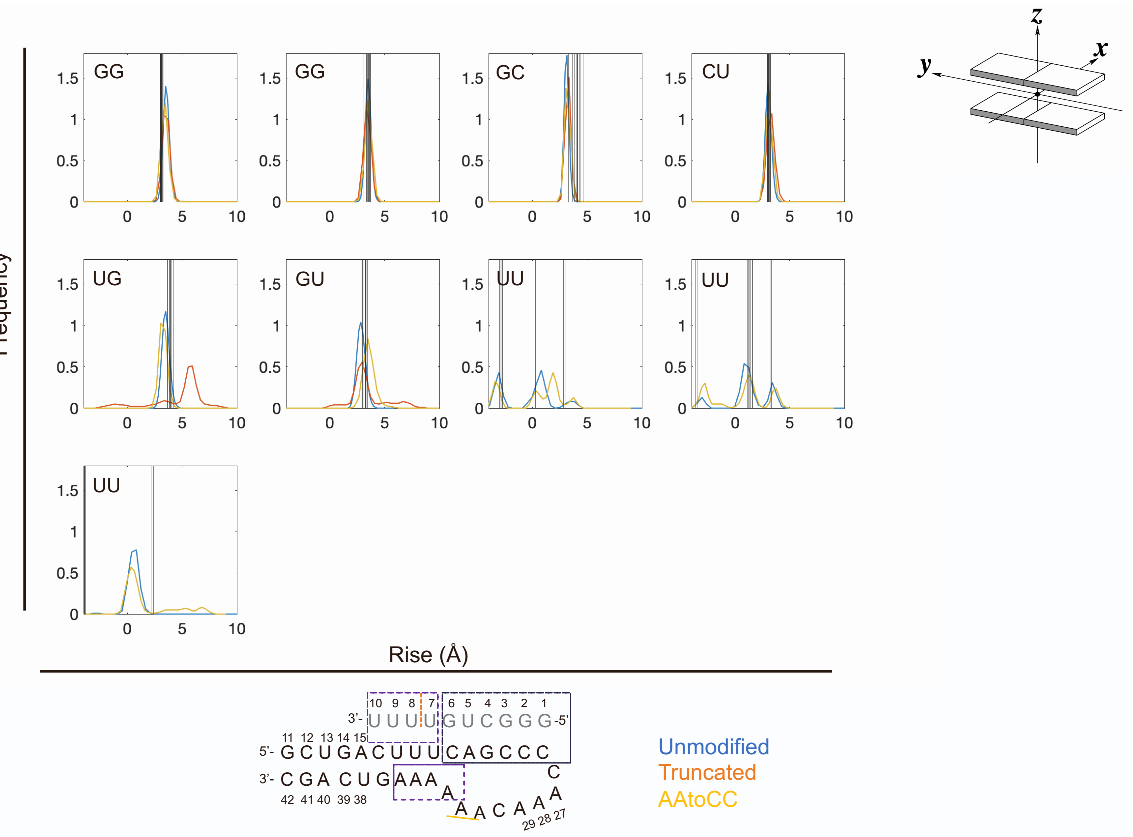

Frequency
GG
GG
GC
CU
UG
GU
UU
UU
UU
Rise (Å)
3'- U U U U G U C G G G -5'
5'- G C U G A C U U U C A G C C C
3'- C G A C U G A A A
Unmodified
Truncated
AAtoCC

D

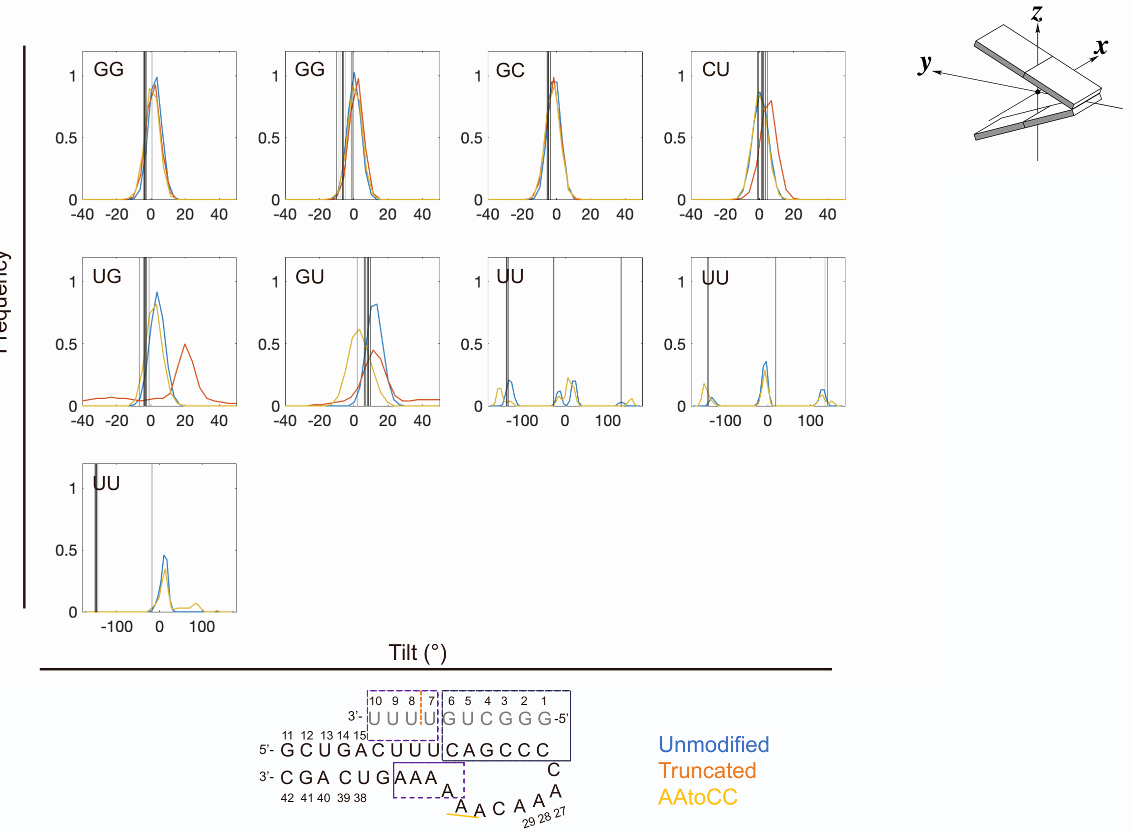

Frequency
GG
GG
GC
CU
UG
GU
UU
UU
UU
Tilt (°)
3'- U U U U G U C G G G -5'
5'- G C U G A C U U U C A G C C C
3'- C G A C U G A A A
Unmodified
Truncated
AAtoCC

E

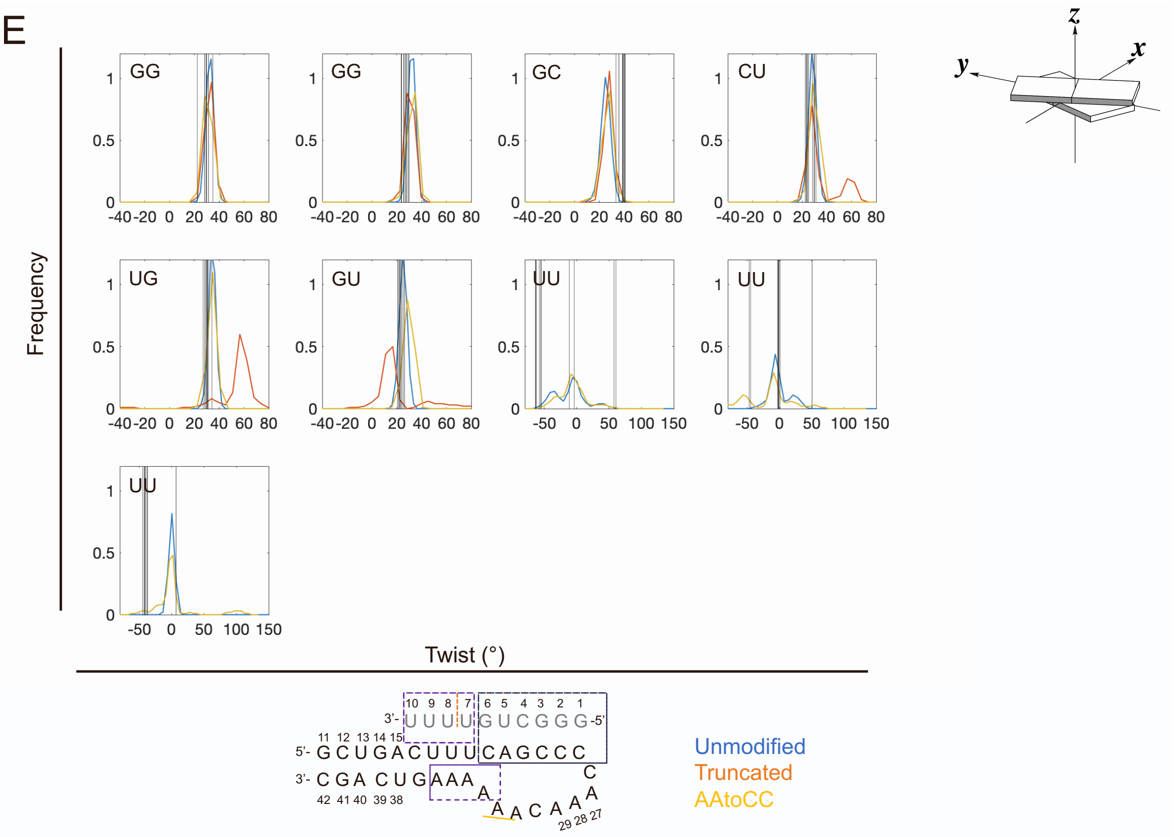

F

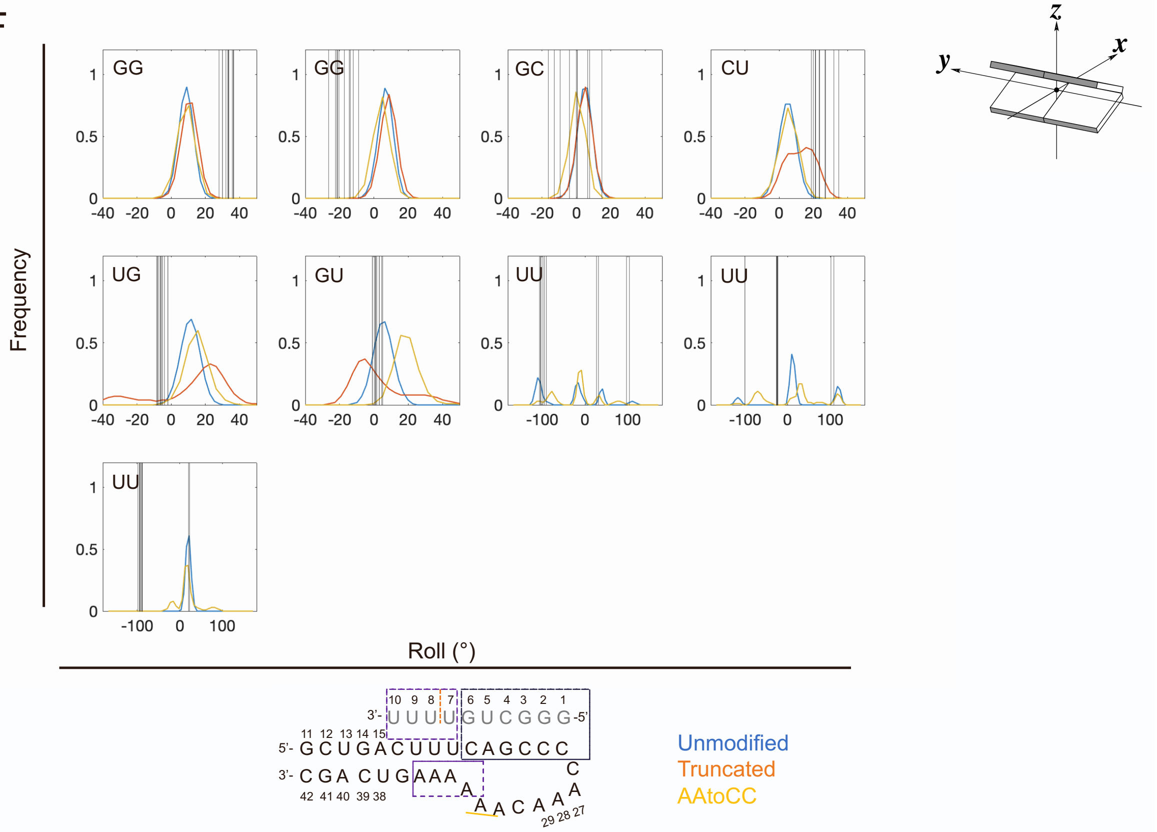

Figure S12: Value distributions of base pair step parameters of the oligo and the hairpin formed between bases 1-6, 20-25 (highlighted with a black rectangle), and bases 7-10, 33-36 (highlighted with a purple rectangle). The b.p. steps for the oligo strand are shown as labels in each plot. Vertical black lines constitute the corresponding values for the NMR models 1-10 (PDB id: 1YMO). **A.** Shift **B.** Slide **C.** Rise **D**. Tilt **E.** Twist **F.** Roll.

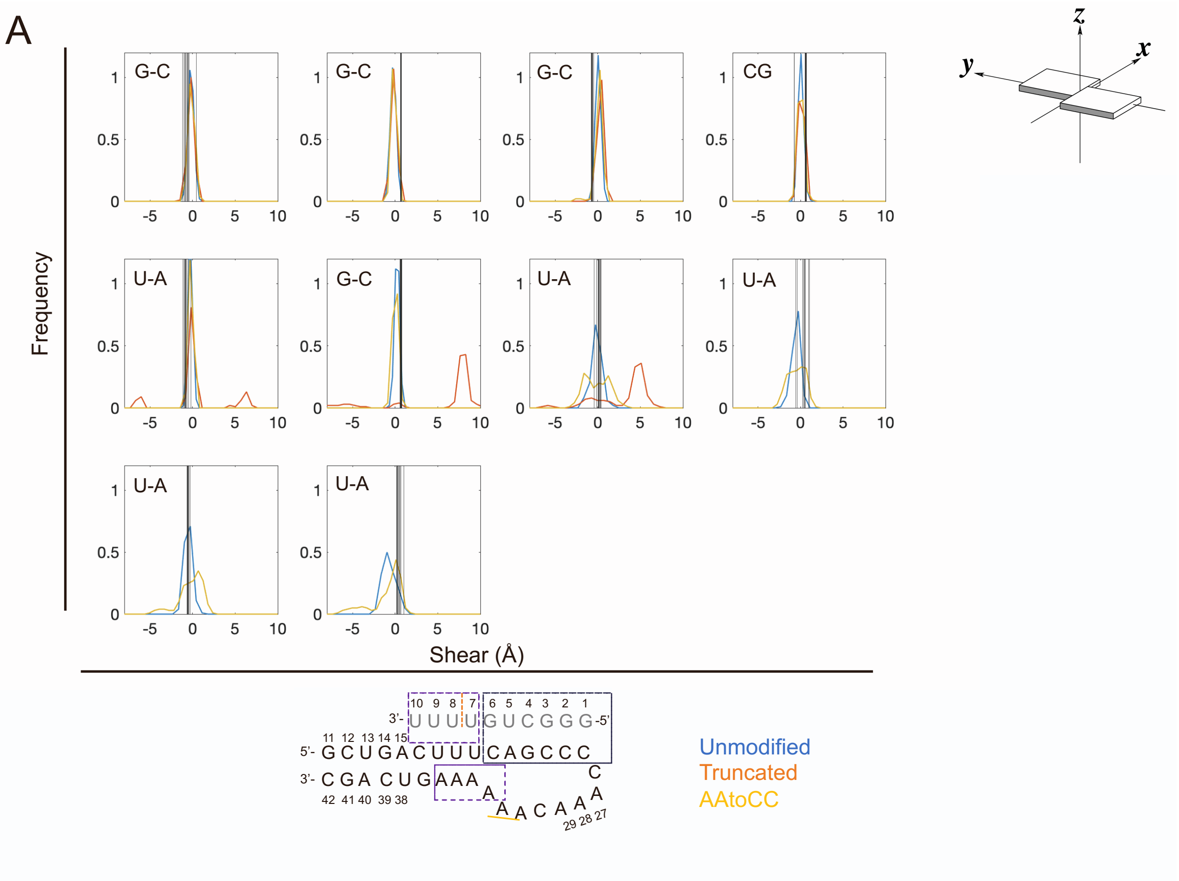
A
Frequency
Shear (Å)
G-C
CG
U-A
Unmodified
Truncated
AAtoCC

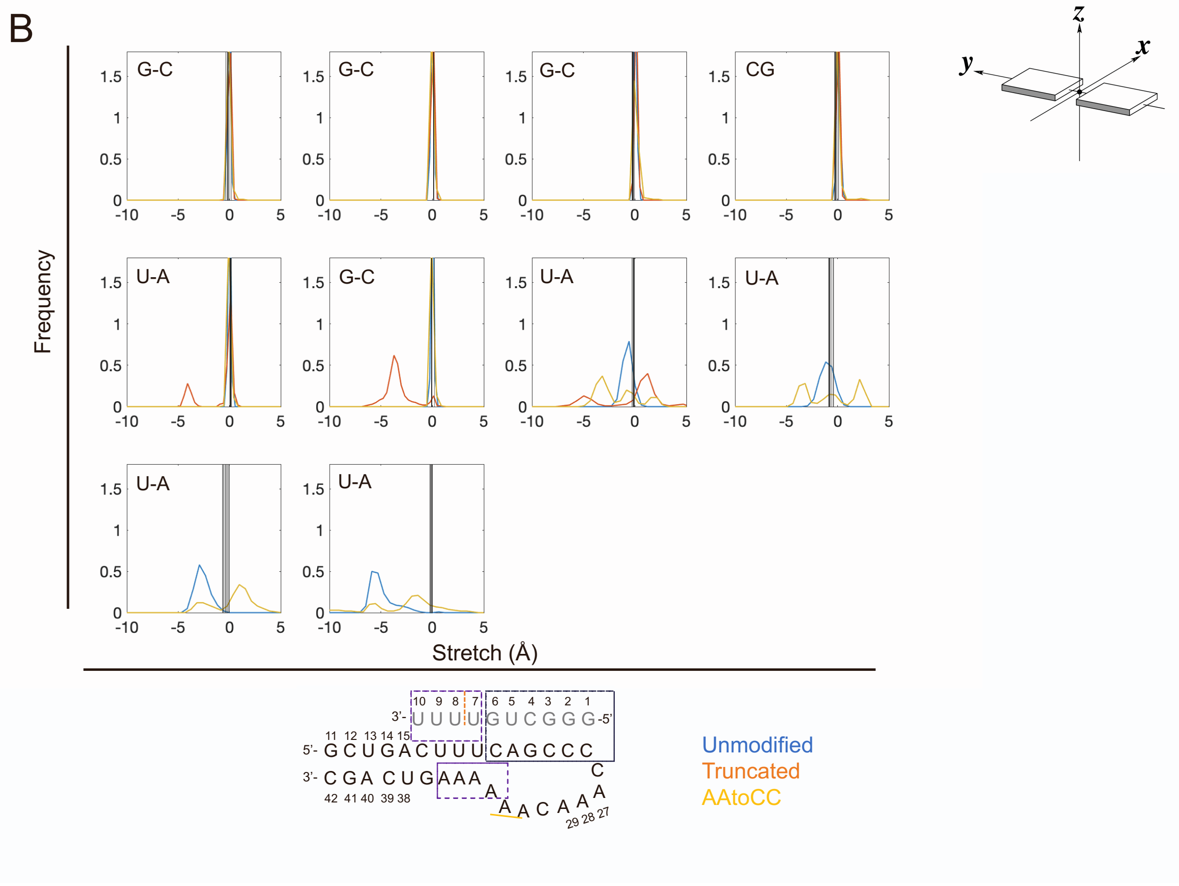
B
Frequency
Stretch (Å)
G-C
CG
U-A
Unmodified
Truncated
AAtoCC

C

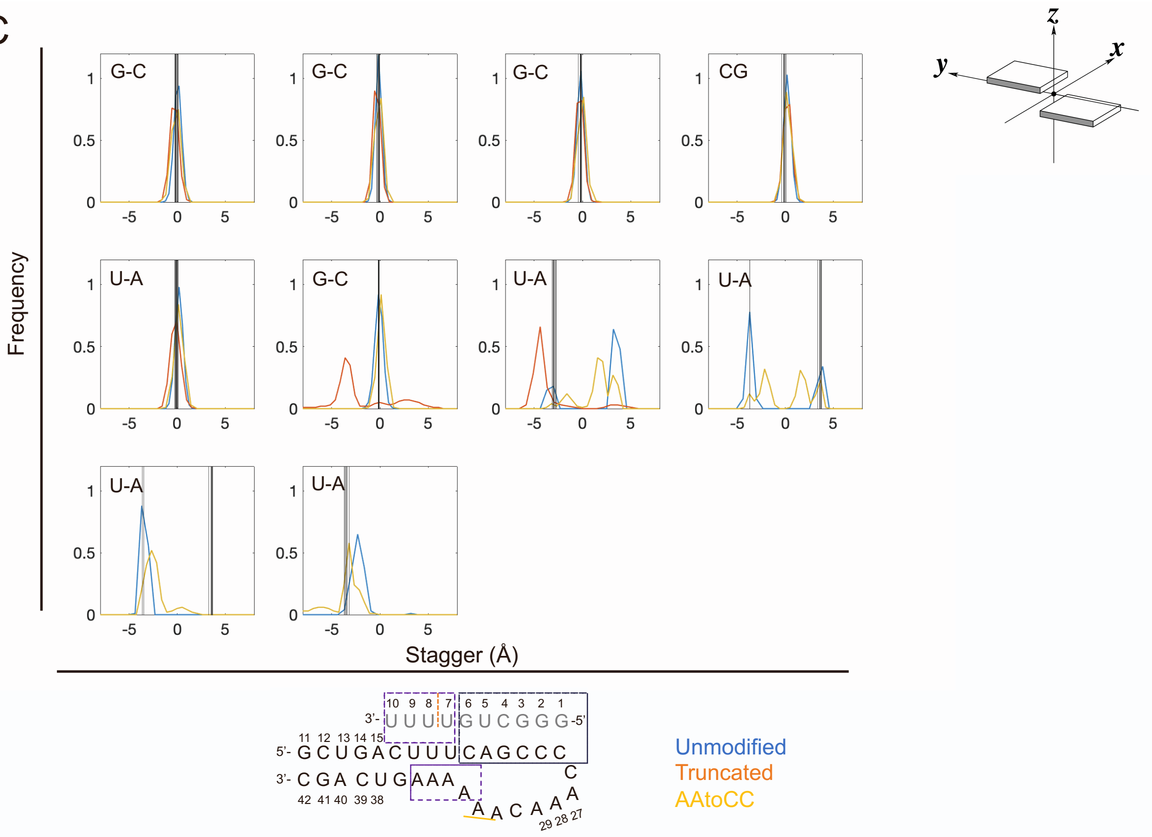

G-C
G-C
G-C
CG
U-A
G-C
U-A
U-A
U-A
U-A
Frequency
Stagger (Å)
z
y
x
Unmodified
Truncated
AAtoCC

D

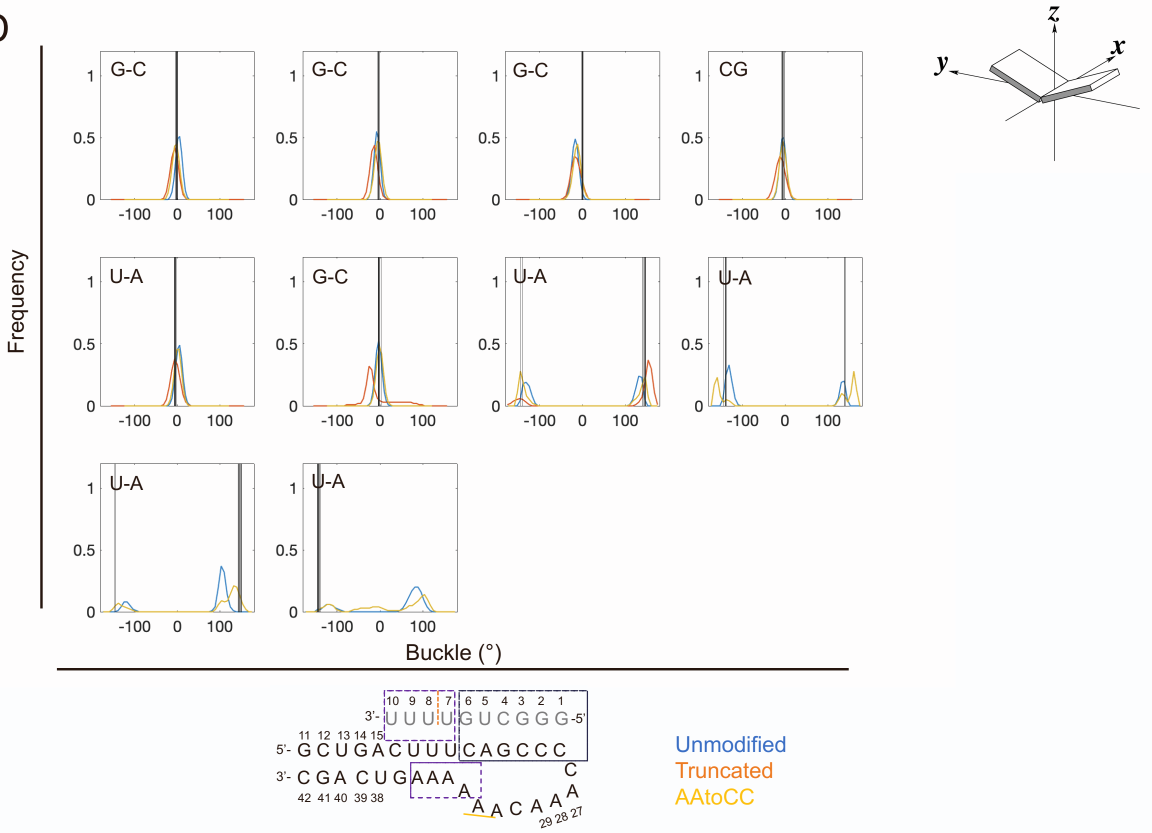

G-C
G-C
G-C
CG
U-A
G-C
U-A
U-A
U-A
U-A
Frequency
Buckle (°)
z
y
x
Unmodified
Truncated
AAtoCC

E

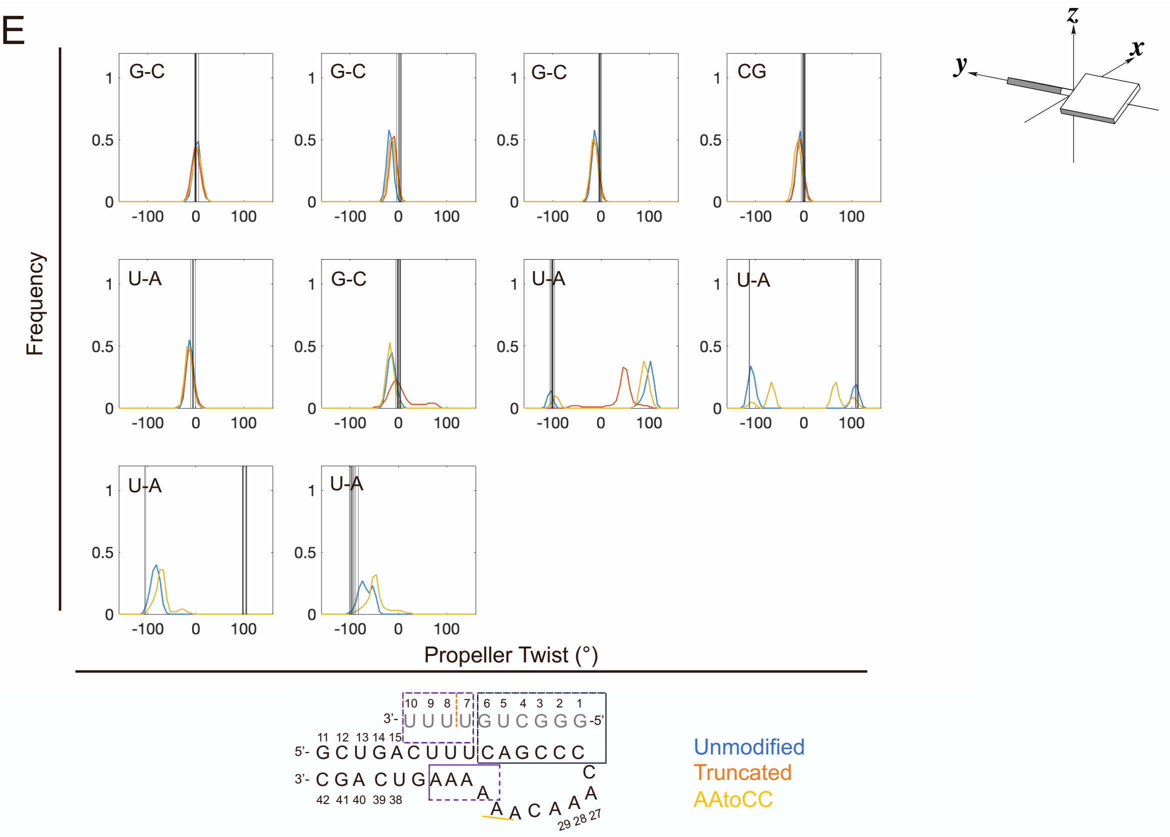

F

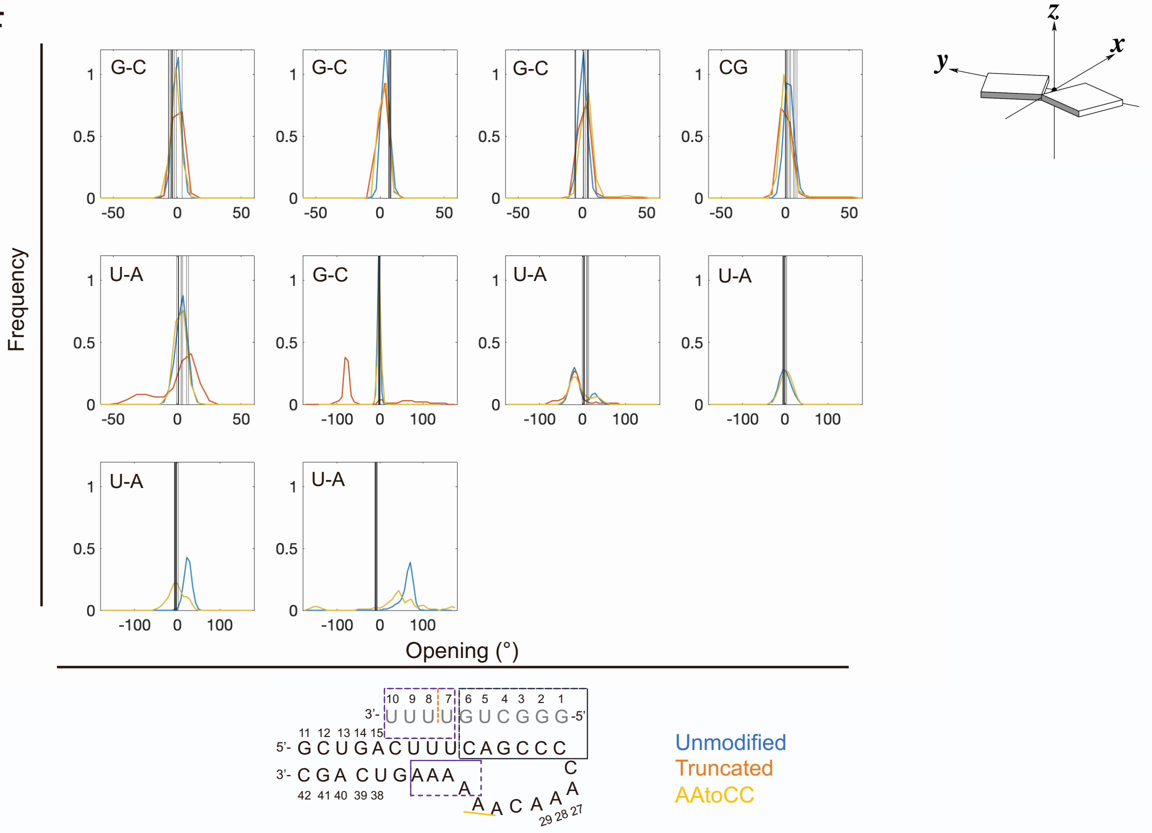

Figure S13: Value distributions of base pair parameters of the oligo and the hairpin formed between bases 1-6, 20-25 (highlighted with a black rectangle) and bases 7-10, 33-36 (highlighted with a purple rectangle). The b.p between the oligo and the hairpin are shown as labels in each plot. Vertical black lines constitute the corresponding values for the NMR models 1-10 (PDB id: 1YMO). **A.** Shear **B.** Stretch **C.** Stagger **D.** Buckle **E.** Propeller twist **F.** Opening.

A

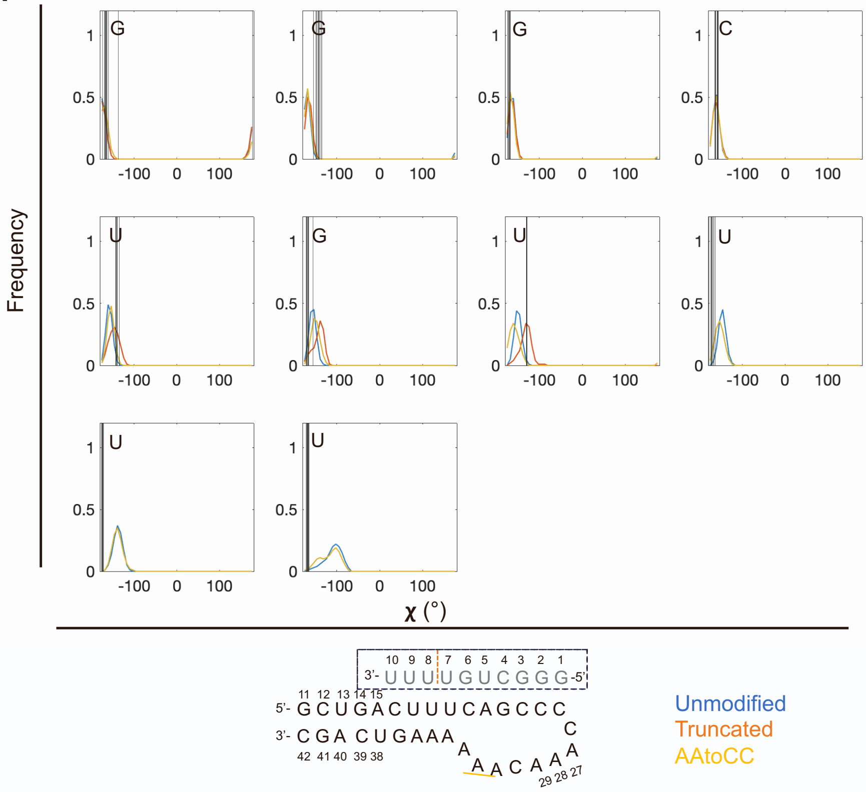
Frequency
χ (°)
3'- U U U U G U C G G G -5'
5'- G C U G A C U U U C A G C C C
3'- C G A C U G A A A
Unmodified
Truncated
AAtoCC

B

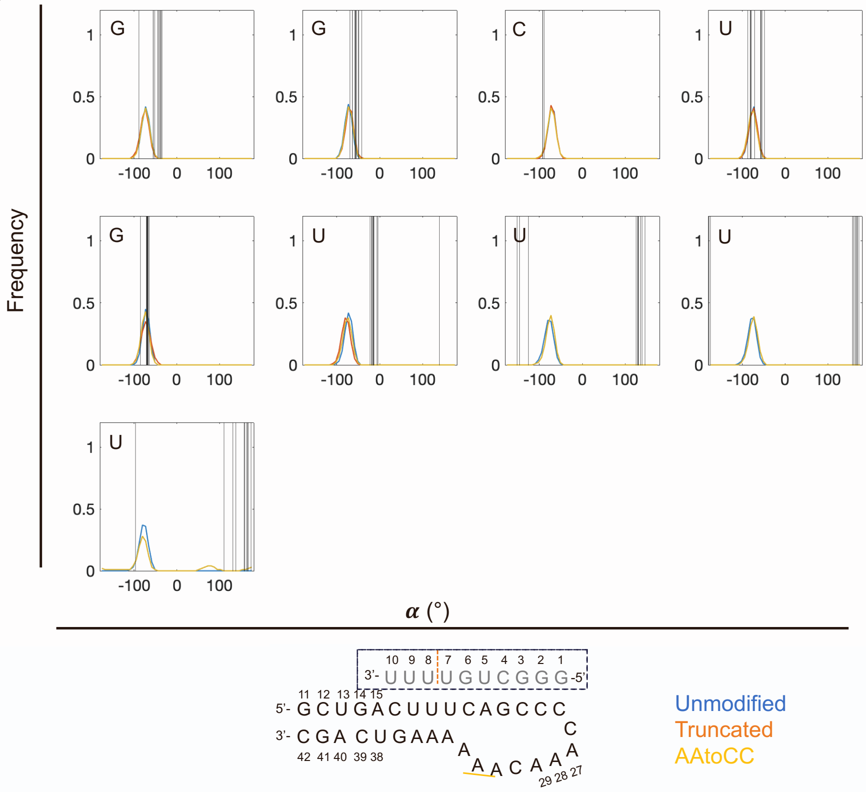
Frequency
α (°)
3'- U U U U G U C G G G -5'
5'- G C U G A C U U U C A G C C C
3'- C G A C U G A A A
Unmodified
Truncated
AAtoCC

C

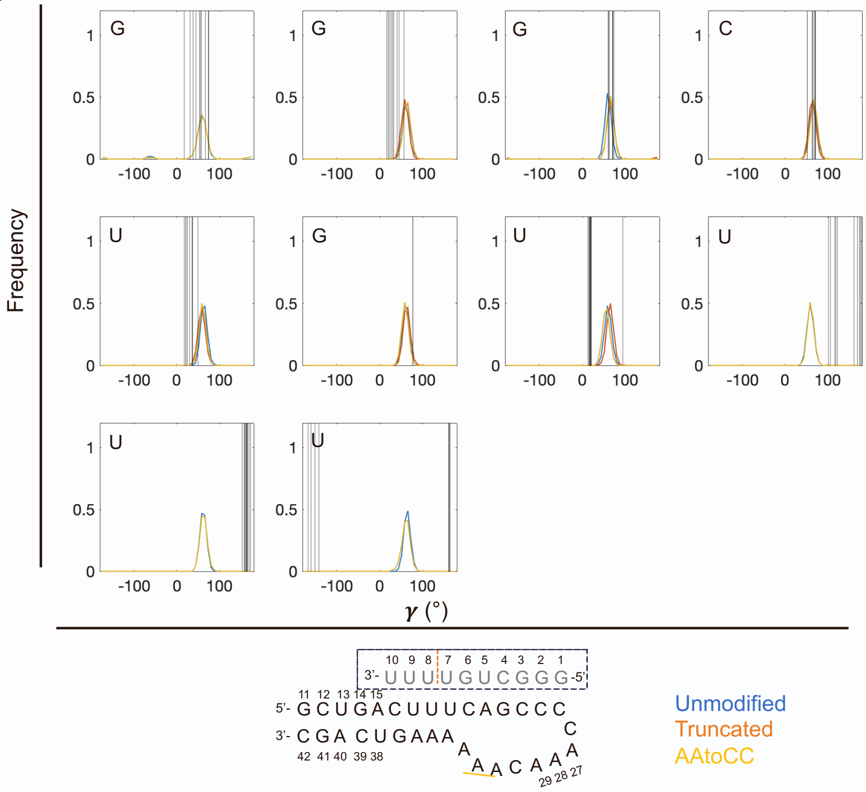
Frequency
G
G
G
C
U
G
U
U
U
U
γ (°)
10 9 8 7 6 5 4 3 2 1
3'- U U U U G U C G G G -5'
11 12 13 14 15
5'- G C U G A C U U U C A G C C C
3'- C G A C U G A A A
42 41 40 39 38
A A A C A A A C
29 28 27
Unmodified
Truncated
AAtoCC

D

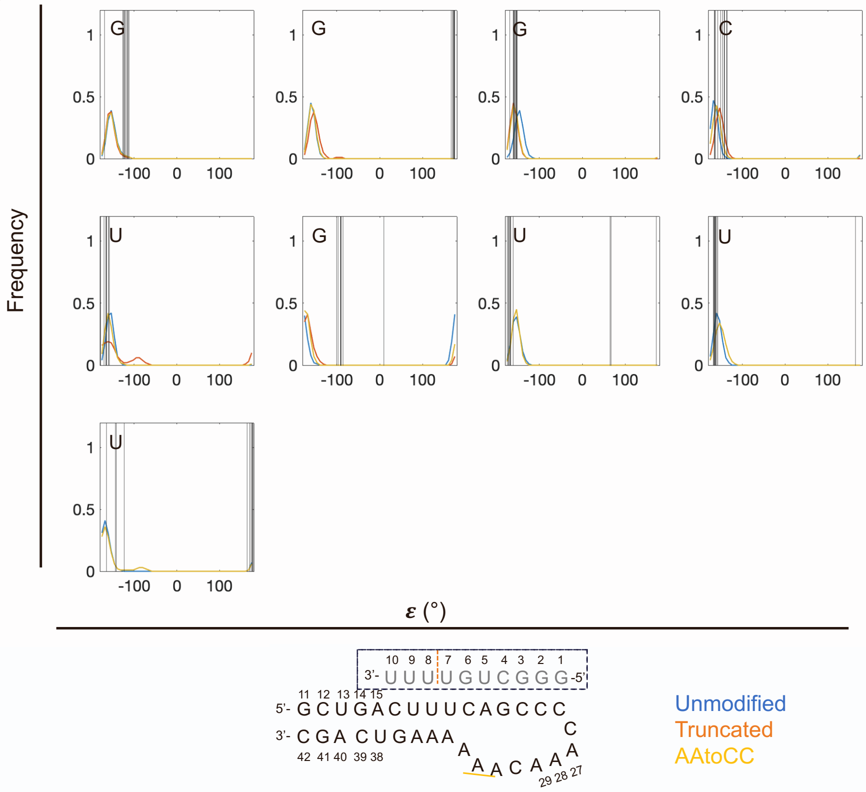
Frequency
G
G
G
C
U
G
U
U
U
ε (°)
10 9 8 7 6 5 4 3 2 1
3'- U U U U G U C G G G -5'
11 12 13 14 15
5'- G C U G A C U U U C A G C C C
3'- C G A C U G A A A
42 41 40 39 38
A A A C A A A C
29 28 27
Unmodified
Truncated
AAtoCC

E

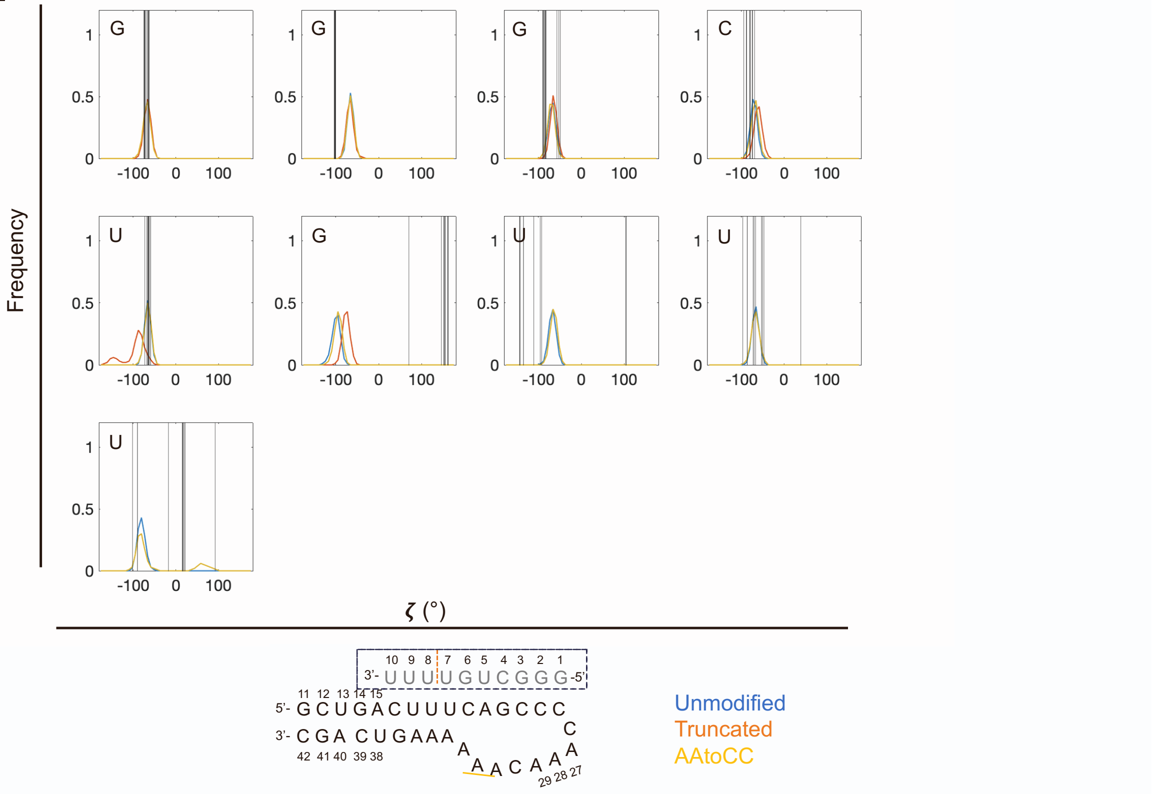

Figure S14: Value distributions of torsional angels for the oligo bases 1-10. Vertical black lines constitute the corresponding values in the NMR models 1-10 (PDB id: 1YMO). **A.** χ **B.** α **C.** γ **D.** ε **E.** ζ.

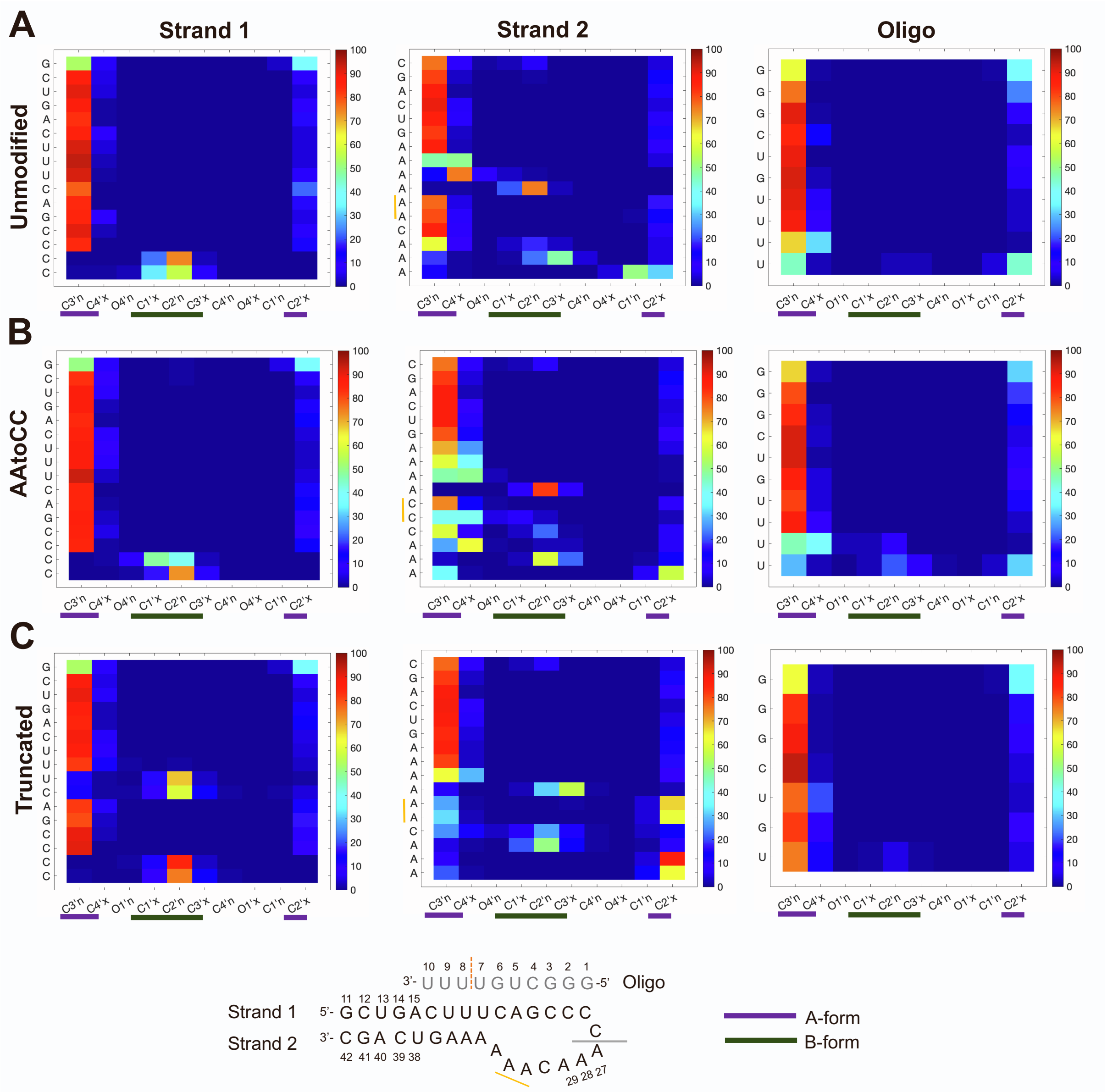

Figure S15: Sugar pucker distributions (%) denoted with a colorbar for bases 11-26 (denoted as "strand 1"), bases 27-42 ("strand 2"), and bases 1-10 ("oligo/ASO"). Conformations typical for the A-form RNA are highlighted with a purple box and conformations typical for the B-form RNA are highlighted with a green box. "n" and "x" are abbreviations for the "endo" and "exo" conformations. **A**. The unmodified; **B**. the mutated; and **C**. the truncated variants.

Supplementary movies legends:

Movie S1: **ASO-hairpin stem interactions of the unmodified system**. Interactions of the ASO 3'-tail with the hairpin stem in the unmodified system.

Movie S2: **ASO-hairpin loop interactions of the unmodified system**. Evolution of interactions of ASO bases U5 and G6 with the hairpin loop of the unmodified system.

Movie S3: **ASO-hairpin loop interactions of the truncated ASO system.** Evolution of interactions of ASO bases 5-7 with the hairpin loop of the truncated ASO system.

Movie S4: **ASO-hairpin loop interactions of the AAtoCC system.** Evolution of interactions of ASO bases U5 and G6 with the hairpin loop of the AAtoCC system.

Movie S5: **ASO-hairpin stem interactions of the AAtoCC system**. Changes in interactions of the ASO 3'-tail with the hairpin stem along the trajectory for the AAtoCC system.

Movie S6: **PC1 of the unmodified system.** Movements corresponding to the principal component 1 of the unmodified system.

Movie S7: **PC2 of the unmodified system.** Movements corresponding to the principal component 2 of the unmodified system.

Movie S8: **PC3 of the unmodified system**. Movements corresponding to the principal component 3 of the unmodified system.

Movie S9: **PC1 of the truncated ASO system.** Movements corresponding to the principal component 1 of the truncated ASO system.

Movie S10: **PC2 of the truncated ASO system.** Movements corresponding to the principal component 2 of the truncated ASO system.

Movie S11: **PC3 of the truncated ASO system.** Movements corresponding to the principal component 3 of the truncated ASO system.

Movie S12: **PC1 of the AAtoCC system.** Movements corresponding to the principal component 1 of the AAtoCC system.

Movie S13: **PC2 of the AAtoCC system.** Movements corresponding to the principal component 2 of the AAtoCC system.

Movie S14: **PC3 of the AAtoCC system.** Movements corresponding to the principal component 3 of the AAtoCC system.
